# Surface antibody changes protein corona both in human and mouse serum but not final opsonization and elimination of targeted polymeric nanoparticles

**DOI:** 10.1186/s12951-023-02134-4

**Published:** 2023-10-14

**Authors:** Sara Capolla, Federico Colombo, Luca De Maso, Prisca Mauro, Paolo Bertoncin, Thilo Kähne, Alexander Engler, Luis Núñez, Ruben Spretz, Gustavo Larsen, Michele Dal Bo, Giuseppe Toffoli, Paolo Macor

**Affiliations:** 1 Experimental and Clinical Pharmacology Unit, Centro di Riferimento Oncologico (CRO) di Aviano, Istituto di Ricovero e Cura a Carattere Scientifico (IRCCS), Aviano, 33081 Italy; 2 Institute for Molecular Systems Engineering and Advanced Materials (IMSEAM), Ruprecht-Karls-Universität Heidelberg, Im Neuenheimer Feld 225, 69120 Heidelberg, Germany; 3 Department of Life Sciences, University of Trieste, via L. Giorgieri n. 5, Trieste, 34127 Italy; 4 Institute of Exptl. Internal Medicine, Medical Faculty, Otto von Guericke University, Magdeburg, 39120 Germany; 5 BioTarget Inc, Chicago, IL USA; 6 Natural Science Department, Concordia University, 7400 Augusta St, River Forest, IL 60305 USA; 7 LNK Chemsolutions LLC, Lincoln, NE USA; 8 Department of Chemical and Biochemical Engineering, University of Nebraska Lincoln, Lincoln, NE USA

**Keywords:** Polymeric nanoparticles, Mouse serum, Human serum, Human plasma, Complement system, Macrophages

## Abstract

**Background:**

Nanoparticles represent one of the most important innovations in the medical field. Among nanocarriers, polymeric nanoparticles (PNPs) attracted much attention due to their biodegradability, biocompatibility, and capacity to increase efficacy and safety of encapsulated drugs. Another important improvement in the use of nanoparticles as delivery systems is the conjugation of a targeting agent that enables the nanoparticles to accumulate in a specific tissue. Despite these advantages, the clinical translation of therapeutic approaches based on nanoparticles is prevented by their interactions with blood proteins. In fact, the so-formed protein corona (PC) drastically alters the biological identity of the particles. Adsorbed activated proteins of the complement cascade play a pivotal role in the clearance of nanoparticles, making them more easily recognized by macrophages, leading to their rapid elimination from the bloodstream and limiting their efficacy. Since the mouse is the most used preclinical model for human disease, this work compared human and mouse PC formed on untargeted PNPs (uPNPs) and targeted PNPs (tPNPs), paying particular attention to complement activation.

**Results:**

Mouse and human serum proteins adsorbed differently to PNPs. The differences in the binding of mouse complement proteins are minimal, whereas human complement components strongly distinguish the two particles. This is probably due to the human origin of the Fc portion of the antibody used as targeting agent on tPNPs. tPNPs and uPNPs mainly activate complement via the classical and alternative pathways, respectively, but this pattern did not affect their binding and internalization in macrophages and only a limited consumption of the activity of the human complement system was documented.

**Conclusions:**

The results clearly indicate the presence of complement proteins on PNPs surface but partially derived from an unspecific deposition rather than an effective complement activation. The presence of a targeting antibody favors the activation of the classical pathway, but its absence allows an increased activation of the alternative pathway. This results in similar opsonization of both PNPs and similar phagocytosis by macrophages, without an impairment of the activity of circulating complement system and, consequently, not enhancing the susceptibility to infection.

**Graphical abstract:**

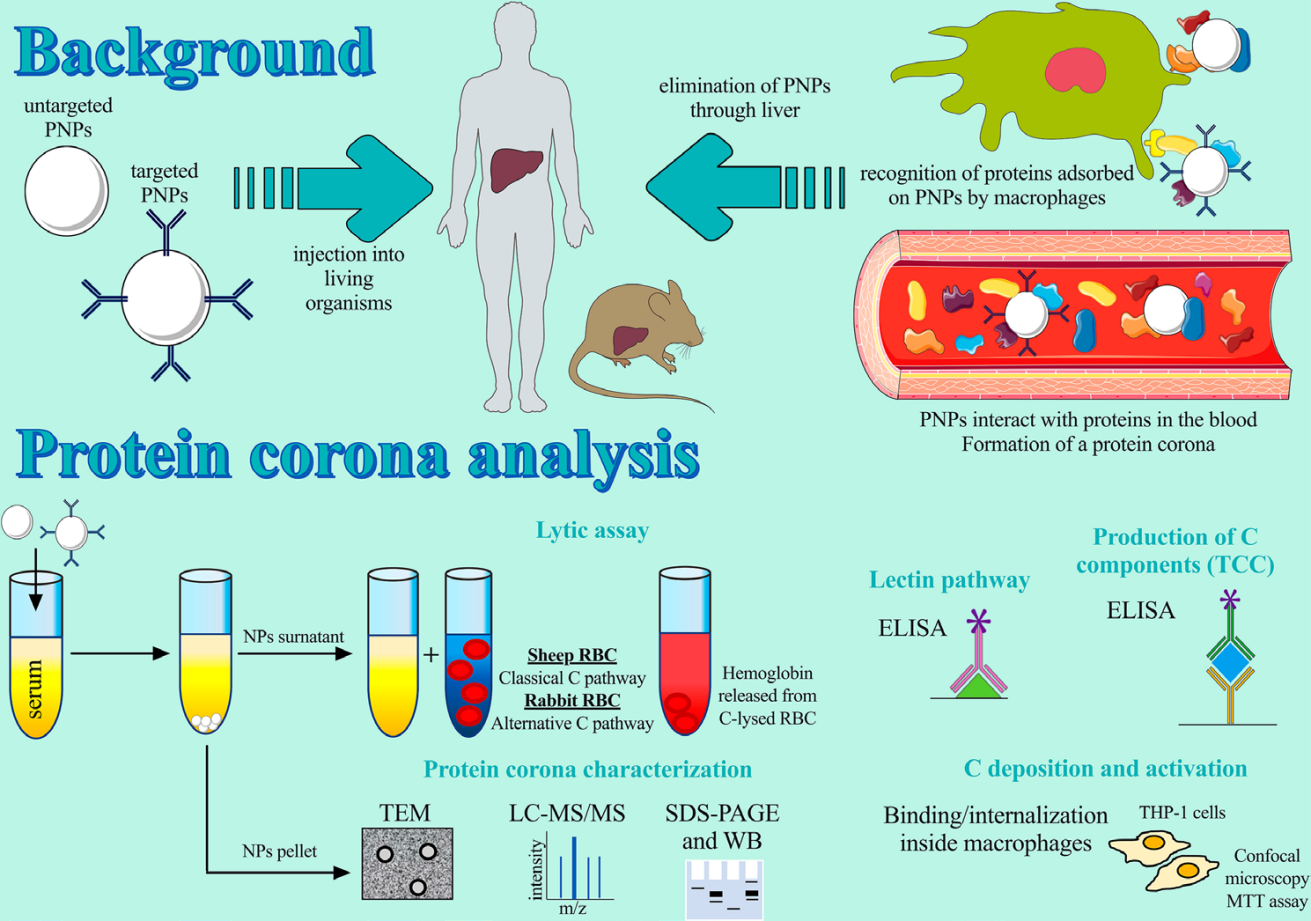

**Supplementary Information:**

The online version contains supplementary material available at 10.1186/s12951-023-02134-4.

## Background

Nanoparticles (NPs) have been extensively studied for diagnostic and therapeutic purposes over the past 30 years. The successful use of NPs in medicine is related to several factors: their high bioavailability, their ability to be loaded with multiple drugs and/or diagnostic tools, the specific delivery of the payload directly to the site of action through surface modifications (e.g. the conjugation with targeting molecules), and, as a consequence, their ability to alter the biodistribution of free drugs, thereby increasing the efficacy of therapies and reducing their toxicity [1]. Among nanocarriers, biodegradable polymeric NPs (PNPs) has gained a lot of attention because of the important improvements made in the field. In fact, biodegradable polymers are exceptional candidates for drug delivery because they are renewable, cost-effective and they are characterized by additional important features that render them more promising as drug delivery systems than other types of NPs: they can be naturally degraded under biological conditions forming non-toxic metabolites, and they can avoid the recognition by immune system cells of the reticuloendothelial system responsible for the elimination of foreign objects further prolonging their circulation time [2].

However, even when the therapeutic efficacy of a nanomedicine has been demonstrated in preclinical and clinical trials, nanomaterials are characterized by poor clinical translatability [3]. A key factor is the dynamic interplay between NPs and thousands of proteins, at different concentrations, present in the blood circulation [4], which leads to the formation of a protein layer, called protein corona (PC), surrounding NPs. The importance of the PC lies in its ability to alter the physiochemical properties of NPs, such as size, shape, surface composition, and charge, as well as functionality, giving them a new biological identity that determines cellular uptake, bioavailability, and toxicity [5, 6]. In particular, the liver is responsible for sequestering more than 95% of injected NPs [7], as this organ is enriched with macrophages that recognize PC proteins [8].

An important group of proteins involved in this process is the complement (C) system. It comprises more than 30 proteins in the blood and is a critical component of innate immunity that rapidly binds on the surface of the targets (opsonization), mobilizing the immune system and coordinating the elimination of the opsonized-target [9]. The similarities between pathogens and NPs, such as size scale and surface chemistry, make the interaction of nanodrugs with the C system not unexpected. Indeed, C has been reported in PC of PNPs, including poly(methyl vinyl ether-co-maleic anhydride) (PVMA) NPs [10], poly(2-methyl-2-oxazoline) (PMOXA)-coated NPs [11], chitosan NPs naked and functionalized with hyaluronic acid and alginate [12] and amine-terminated poly(amido) amine (PAMAM) dendrimers [13].

The C system can be activated by three downstream pathways, depending on which proteins recognize NPs: the binding of C1q in the C1q-C1r-C1s complex (called C1) to an antigen-antibody complex initiates the classical pathway; the alternative pathway is triggered by spontaneous hydrolysis of the third protein (C3) of the cascade; binding of carbohydrates on the surface of pathogens or the plasma protein mannose-binding lectins (MBL) to NPs trigger the lectin pathway. After activation, all pathways end with the same common step: C3 is cleaved, producing the opsonic fragments C3b and an anaphylatoxin (C3a). Opsonins (e.g., C3b but also inactive C3b, IgG, etc.) are rapidly recognized by receptors expressed on macrophages, resulting in fast clearance of NPs, whereas anaphylatoxins (C3a, C4a, and C5a) are biologically active fragments that trigger various immunological responses such as inflammation, chemotaxis, cardiopulmonary distress [14], activation of the adaptive immune system by stimulating B-cell response, activation of dendritic cells and T lymphocytes, and hypersensitivity reactions [9].

A complete characterization of the molecular interactions between C system and NPs is pivotal in their development because its activation influences their biodistribution, stability and biological action.

To attenuate the opsonization of NPs, they are often modified with hydrophilic polymers such as polyethylene glycol (PEG), which suppresses the nonspecific interaction between NPs and proteins, by forming a protective layer, and prolongs the retention time in blood [15]. Although several studies suggest that PEGylation of NPs, which include silica NPs [11] and liposomes [16], cannot completely prevent the opsonization of NPs by C components, leading to their rapid elimination by macrophages, PEG still shows a lower immunological profile compared with other polymers [11]. Given that the presence of C proteins in PC does not necessarily contribute to the rapid elimination of NPs by macrophages and the paucity of studies on PEGylated polymeric NPs, this study aims to assess the extent of C deposition and activation on polylactic acid (PLA)-PEG-poly(ε-caprolactone) (PCL) NPs as well as the modifications caused by a surface targeting antibody.

## Methods

### Synthesis and characterization of PNPs

The chemicals used to prepare the PNPs were reagent grade or better and include biodegradable carboxylic acid-terminated polymers (PLA-b-PEG-COOH and PCL-COOH). PNPs were prepared with an expected average diameter of 250 nm and under class 100 cleanroom conditions using a proprietary electrohydrodynamic technology (BioTarget Inc., Chicago, IL, USA; LNK Chemsolutions LLC, Lincoln, Nebraska, USA). The method of producing these types of PNPs has been described in detail in patent number US-2,013,017,148-A1. Briefly, an organic solution containing all the components was processed using this technology, resulting in a dry collection of the specified PNPs (1.66 mg/mL of polymers). The collected material was then harvested in an aqueous buffer solution to obtain a stable suspension. The targeting molecule (anti-CD20 antibody from the clinic, 12 µg/mL) was conjugated on the surface of the NPs using its primary amines, which can react with polymers functionalized with N-Hydroxysuccinimide (NHS) groups. The prepared PNPs were resuspended in phosphate-buffered saline (PBS) pH 7.4 containing 0.3% bovine serum albumin (BSA).

The core fluid solution was prepared by dissolving Chlorambucil (CLB) and Hydroxychloroquine sulfate (HCQ) in an aqueous solution of propanol and isopropanol as described in the patent.

To label the PNPs with fluorescein-5-isothiocyanate (FITC), 1 mg polymer was incubated with 50 µg FITC (Sigma Aldrich, Milan, Italy) diluted in dimethyl sulfoxide (DMSO) for 4 h at 4 °C under rotation. Then, 50 mM NH_4_Cl 1 M pH 6.5 was added to the solution to block the labeling reaction. Finally, the PNPs were dialyzed in PBS. Final concentration of FITC: 0.4 mg/mL.

### Formation of the PC on PNPs

Targeted (tPNPs, 62.3 µg polymer) and untargeted PNPs (uPNPs, 62.3 µg polymer) were washed three times at 5900 g for 1.5 min to eliminate the excess of albumin used in the storage solution, and incubated with serum from healthy C57/BL mice (mouse serum, MS, pool of 5 mice) or human serum (HS, pool of 20 donors) diluted 1:2 in PBS containing 0.15 mM CaCl_2_ and 0.5 M MgCl_2_, or human plasma (HP, pool of 20 donors) diluted 1:2 in PBS containing 20 mM ethylenediaminetetraacetic acid (EDTA) for 30 min with shaking (800 rpm) at 37 °C. At the end of incubation, PNPs were washed three times by centrifugation (5900 g for 1.5 min) to remove the excess of unbound serum proteins. The resulting pellet of PNPs and the supernatant were used to study the PC formation on the PNPs and the deposition of C-cascade proteins.

For the study of binding and internalization of PNPs in macrophages, PNPs were washed three times and incubated with HS or HP for 30 min with shaking (800 rpm) at 37 °C. The suspension of PNPs in HS or HP was added directly to cells maintained in a serum-free medium.

### TEM

Transmission electron microscopy (TEM) analysis was used to measure the change in size of PNPs before and after incubation with HS. PNPs were diluted 1:50 in ultrapure H_2_O and deposited on a carbon foil supported on copper microgrids. Images were acquired using a Philips CM100 TE microscope (Philips Research, Eindhoven, The Netherlands) at 80 kV.

### Liquid chromatography-mass spectrometry (LC-MS/MS)

Sample preparation for MS was performed via On-Beads digestion for the best analytical sensitivity. In brief, nanoparticles, previously incubated with HS or MS, were resuspended in 50 mM NH_4_HCO_3_, pH 8.0, and subsequently incubated with 1 mM dithiothreitol at 56 °C for 45 min. Afterwards, reduced cysteine residues were ß-methylthiolated by addition of 5 mM methyl methanethiosulfonate at room temperature for 30 min. Proteins were digested by adding 0.5 µg trypsin (Trypsin Gold, Promega, Milan, Italy) at 37 °C overnight. Generated peptides were gathered by collecting the supernatants combined with two washing steps of the beads using 50 µl of 25 mM NH_4_HCO_3_ for each wash. All supernatants of a sample were pooled and dried down in a vacuum centrifuge. The peptides were redissolved in 5 µl 0.1% trifluoroacetic acid (TFA) and purified on ZIP-TIP, C18-nanocolumns (Millipore, Billerica, USA). The peptides were eluted in 7 µl of 70% (v/v) acetonitrile (ACN) and subsequently dried in a vacuum centrifuge.

LC-MS/MS was performed on a hybrid dual-pressure linear ion trap/orbitrap mass spectrometer (LTQ Orbitrap Velos Pro, Thermo Scientific, San Jose, CA, USA) equipped with an EASY-nLC Ultra HPLC (Thermo Scientific, San Jose, CA, USA). The peptide samples were dissolved in 10 µl of 2% ACN/0.1% TFA and fractionated on a 75 μm I.D., 25 cm PepMap C18-column, packed with 2 μm resin (Dionex, Germany). Separation was achieved by applying a gradient from 2% ACN to 35% ACN in 0.1% FA over a 150 min gradient at a flow rate of 300 nl/min. The LTQ Orbitrap Velos Pro MS exclusively used CID-fragmentation when acquiring MS/MS spectra consisted of an orbitrap full MS scan followed by up to 15 LTQ MS/MS experiments (TOP15) on the most abundant ions detected in the full MS scan. The essential MS settings were as follows: full MS (FTMS; resolution 60.000; m/z range 400–2000); MS/MS (Linear Trap; minimum signal threshold 500; isolation width 2 Da; dynamic exclusion time setting 30 s; singly charged ions were excluded from selection). Normalized collision energy was set to 35%, and the activation time was set to 10 ms.

Raw data processing, protein identification and PTMs assignment was performed with the *de novo* sequencing algorithms of PEAKS Studio 8.0 (Bioinformatics Solutions). The false discovery rate was set to < 1%.

The abundance values are arbitrary values, generated by mass spec peak integration.

### SDS-PAGE and western blot analyses

The pellet of PNPs, obtained after centrifugation, was resuspended directly in reducing sample buffer (Tris 0.35 M pH 6.8, 30% glycerol, 10% SDS, 0.1% bromophenol blue, 5% 2-mercaptoethanol), boiled for 5 min, and loaded into a sodium dodecyl sulphate – polyacrylamide gel electrophoresis (SDS-PAGE). Proteins were visualized by Coomassie staining: the gel was stained for 2 to 3 h with a solution of 0.1% Coomassie Brilliant blue G250 in 30% methanol and 10% acetic acid, and proteins were visualized after destaining with 30% methanol and 10% acetic acid. For Western blot (WB) analysis, PNPs were incubated with HS or HP, or MS, centrifuged and loaded into a SDS-PAGE as described, proteins were transferred to a nitrocellulose blotting membrane (Thermo-Fischer Scientific, Waltham, Massachusetts, USA). Primary antibodies such as anti-mouse C1q (Hycult Biotech, The Netherlands, 1:400), anti-mouse C3 (Cappel, 1:5,000), and anti-mouse C9 (gift from Prof. Mohamed Daha, 1:250) were used to detect mouse C components. As a secondary antibody for the detection of C1q and C9, goat anti-rabbit antibody conjugated with alkaline phosphatase (AP) (Sigma Aldrich, Milan, Italy, 1:20,000) was used after preincubation with 1 µg/mL mouse IgG (Sigma Aldrich, Milan, Italy) to remove unspecific binding, whereas C3 deposit was detected using an AP-conjugated donkey anti-goat IgG antibody (Novex, Thermo-Fischer Scientific, Waltham, Massachusetts, U.S, 1:4,000). Mouse IgG and IgM deposition was detected with goat anti-mouse IgG (1:500) and IgM (1:1,000) antibodies, respectively, from Meloy (Springfield, Virginia, USA). An AP-conjugated donkey anti-goat IgG antibody (Novex, Thermo-Fischer Scientific, Waltham, Massachusetts, USA, 1:4,000) was used as secondary antibody.

For the human proteins, anti-human C1q (1:400), C3 (1:8,000), C6 (1:500), C7 (1:5,000), and C9 (1:250) antibodies were purchased from Quidel (San Diego, CA, USA), whereas mouse anti-human IgM was purchased from Vector (Burlingame, CA, USA, 1:1,000). Detection of these antibodies was performed with a secondary AP-conjugated donkey anti-goat antibody (Novex, Thermo-Fischer Scientific, Waltham, MA, USA, 1:4,000). AP-conjugated anti-human IgG antibody was purchased from Jackson ImmunoResearch Europe (UK, 1:2,000).

For WB analyses, all antibodies were incubated in 2% milk for 1 h at room temperature. Three washes were performed to remove excess unbound antibody. Substrate solution for AP was prepared by dissolving 5-bromo-4-chloro-3’-indolyl phosphate p-toluidine (BCIP) and nitroblue tetrazolium chloride (NBT) in AP buffer (0.1 M NaCl, 0.1 M Tris-HCl pH 9.5, 0.005 M MgCl_2_) according to the manufacturer’s instructions (Thermo-Fischer Scientific, Waltham, Massachusetts, USA).

### Evaluation of C activation

Lytic assays were performed by washing (3 times at 5900 g for 1.5 min) and incubating PNPs (13 µg polymers) with 100 µL HS for up to 8 h at 37 °C with shaking (800 rpm). The PNPs were then centrifuged, and the supernatant was incubated with 1% sheep or rabbit red blood cells (RBCs) diluted in C fixation diluent (NaCl 0.142 M, Na-5-5-diethilbarbiturate 5 mM, MgCl2 0.5 mM, CaCl2 0.15 mM, gelatin 0.05%, NaN3 0.01%, pH 7.4) for 30 min to activate the classical or the alternative C pathway, respectively. Activation of both pathways was checked by preparing a standard curve using HS as positive control. The reaction was then blocked by the addition of 20 mM EDTA diluted in PBS. Lysed RBCs was measured using a spectrophotometer at OD = 415 nm (Infinite M200Pro plate reader (Tecan Italia S.r.l., Milan, Italy) after centrifugation at 12,000 g for 1 min. The percentage of C activity was determined by the following operation: % of C activity = [(OD of sample – OD background) / (OD total lysis – OD background)] x 100 [17].

The ELISA kit was used to determine the residual activity of the lectin pathway after serum incubation with PNPs. WIESLAB® Complement System kits (Lundavägen, Malmö, Sweden) were used according to the procedures described by the manufacturers.

ELISA was also used to quantify the formation of the terminal C complex (TCC) after serum incubation with PNPs, as previously described [18]. Briefly, TCC concentration was determined in the supernatant after centrifugation of serum-incubated particles (5900 g) by coating the wells with the anti-C9 monoclonal antibody aE11 (Quidel, San Diego, CA, USA) which recognizes the C9 naoantigens in TCC, and revealing the reaction using a biotinylated anti-human C5 antibody (Quidel, San Diego, CA, USA), AP-conjugated streptavidin (Sigma Aldrich, Milan, Italy) and p-nitrophenyl phosphate (Sigma Aldrich, Milan, Italy). The concentration of TCC was obtained interpolating the absorbance values measured using a spectrophotometer at OD = 405 nm (Infinite M200Pro plate reader (Tecan Italia S.r.l., Milan, Italy) to a standard curve of serum TCC.

### Binding and internalization of PNPs in macrophages

To determine the binding and the internalization of PNPs in macrophages, human monocytes (THP-1 cells) were cultured in RPMI 1640 (Sigma Aldrich, Milan, Italy) supplemented with 10% fetal bovine serum (Thermo Scientific, Waltham, MA, USA) and 1% penicillin/streptomycin (Sigma Aldrich, Milan, Italy) and maintained at 37 °C and 5% CO_2_ humidified atmosphere. Monocytes were differentiated into macrophages by stimulating them with phorbol-12-myristate-13-acetate (PMA, 100 ng/mL, Sigma Aldrich, Milan, Italy) for 72 h. Cells (1 × 10^6^) were then washed and incubated for 1 h with HS- or HP-PNPs in serum-free medium (final serum concentration: 10%). Binding of PNPs was assessed by confocal microscopy (Eclipse te300, Nikon Corporation, Tokyo, Japan) and the fluorescence was quantified using Fiji software [19, 20]. Cell nuclei were labeled with 4′,6-diamidino-2-phenylindole (DAPI, Sigma Aldrich, Milan, Italy) for 5 min at room temperature, while membranes were labeled with Vybrant™ DiI cell-labeling solution (Thermo Fisher Scientific, Waltham, MA, USA) for 20 min at room temperature. At the end of the incubation steps, cells were washed three times with PBS for 15 min. Internalization of PNPs was performed using HCQ-CLB-loaded PNPs. After incubating the cells with HS- or HP-PNPs for 1 h, the macrophages were washed to remove the excess unbound PNPs and grown in complete medium for 48 h. The percentage of live cells was measured using the 3-(4,5-dimethylthiazol-2-yl)-2,5-diphenyl-2 H-tetrazolium bromide (MTT) assay. Briefly, MTT (0.5 mg/mL, Sigma Aldrich, Milan, Italy) was added to the cells and incubated at 37 °C for 4 h. The cells were then centrifuged to remove the supernatant and the formazan crystals formed were dissolved in DMSO. The signal was measured at 570 nm using the Infinite M200Pro plate reader (Tecan Italia S.r.l., Cernusco sul Naviglio, Milan, Italy).

### Statistical analysis

LC-MS/MS data were presented with boxplots; line inside box indicates the position of the median. LC-MS/MS, ELISA data were expressed as mean ± standard error mean (SEM) and analyzed by the two-tailed Student’s t-test to compare two paired groups of data. C activation, binding and internalization data were expressed as mean ± SEM and analyzed by one way ANOVA test to compare three or more groups of data. P-values < 0.05 or less were considered statistically significant.

## Results

### tPNPs bound a higher number of human than murine proteins

**Fig. 1:**
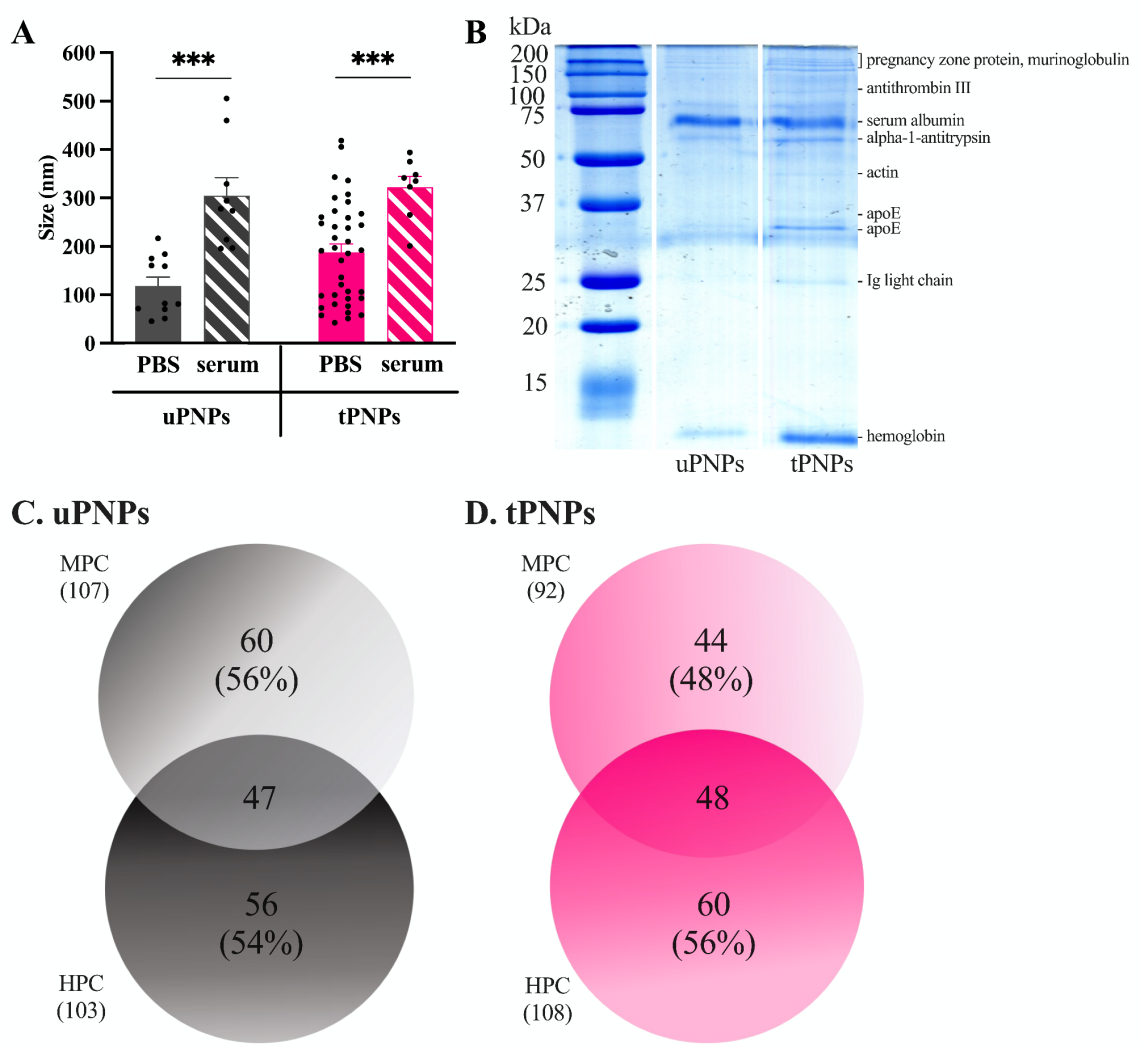
Formation of mouse and human protein corona on PNPs. **A**. Detection of protein corona formation by TEM analysis of PNPs before and after incubation with serum. Data are represented as mean ± SEM and analyzed by the two-tailed Student’s t-test. Black spots represent single measurements. *** p-value <0.001. **B**. Deposition of proteins on PNPs was also detected by SDS-PAGE and Brilliant Blue staining. The identity of the bands was determined by LC-MS/MS analysis and indicated on the right side of SDS-PAGE. The number of different proteins detected on uPNPs (**C**) and tPNPs (**D**) was obtained by LC-MS/MS analysis and reported in a Venn diagram. PBS: phosphate buffered saline; PNPs: polymeric nanoparticles; uPNPs: untargeted PNPs; tPNPs: targeted PNPs; MPC: mouse protein corona; HPC: human protein corona

Two different types of PNPs were produced for this study: untargeted PNPs (uPNPs) and targeted PNPs (tPNPs). Both nanostructures are made of PEG, PCL, and PLA; the difference between them is the conjugation of a human anti-CD20 antibody on the surface of the tPNPs, a targeting agent that is absent on the uPNPs. To avoid aggregation, PNPs were stored in 0.3% BSA, that was mainly removed through centrifugation before incubation with biological fluids. The PNPs have a similar diameter (uPNPs vs. tPNPs 118 ± 61 nm vs. 189 ± 104 nm), taking in consideration the presence of antibodies on the surface, but after incubation with HS their size increases significantly by ~ 150 nm (uPNPs in PBS vs. HS: 118 ± 61 nm vs. 305 ± 111 nm, p-value 0.0007. tPNPs in PBS vs. HS: 189 ± 104 nm vs. 323 ± 62 nm, p-value 0.0002; Fig. 1A).

The increase of the dimension of both PNPs types after incubation with serum is due to both the formation of a PC surrounding the PNPs and the growth of the PNPs themselves, as shown by the TEM analysis (Additional File 1: Supplementary Fig. 1A and 1B). This pattern suggests a lack of stability of the nanocarriers in biological fluids, assuming a loss of function of the PNPs in vitro and in vivo. However, our past studies widely demonstrated the safety and efficacy of PNPs in mouse models of human cancers [21–24] and rheumatoid arthritis [25].

The formation of a PC surrounding the PNPs was confirmed by SDS-PAGE in which protein bands were visible after Coomassie Brilliant Blue staining, indicating the adsorption of serum proteins. PC was enriched in low molecular weight (MW) proteins (e.g., MW < 15 kDa, hemoglobin) and high MW (e.g., ~ 60 kDa, albumin), as suggested by the presence of more intense bands. Other visible bands represent Ig light chains (25 kDa), apoE (33 kDa), actin (45 kDa), alpha-1-antitrypsin (52 kDa), antithrombin III (~ 100 kDa), and pregnancy zone proteins (> 200 kDa) (Fig. 1B). After incubation of the PNPs with HS or MS, the adsorbed proteins were identified by liquid chromatography-mass spectrometry (LC-MS/MS). Almost the same number of proteins were adsorbed to uPNPs; in fact, 107 and 103 proteins were detected in the murine protein corona (MPC) and the human protein corona (HPC), respectively. Among these, 56% of the murine proteins and 54% of the human proteins were uniquely adsorbed to uPNPs (Fig. 1C).

The presence of the human antibody on the tPNPs prevented the binding of some murine proteins; in fact, the MPC consisted of a smaller number of proteins than the HPC (mouse vs. human proteins: 92 vs. 108). This pattern can be explained by the species-specific interactions that an antibody can form with proteins in the serum of the same parent organism. Therefore, the naked polymer-forming uPNPs could interact indiscriminately with murine and human proteins, while the human antibody conjugated to the tPNPs could favor adsorption of some human proteins. As a result, the percentage of unique proteins increased, accounting for 48% of MPC compared to 56% of HPC (Fig. 1D).

### Particles bind preferentially negatively charged and low-MW proteins

**Fig. 2:**
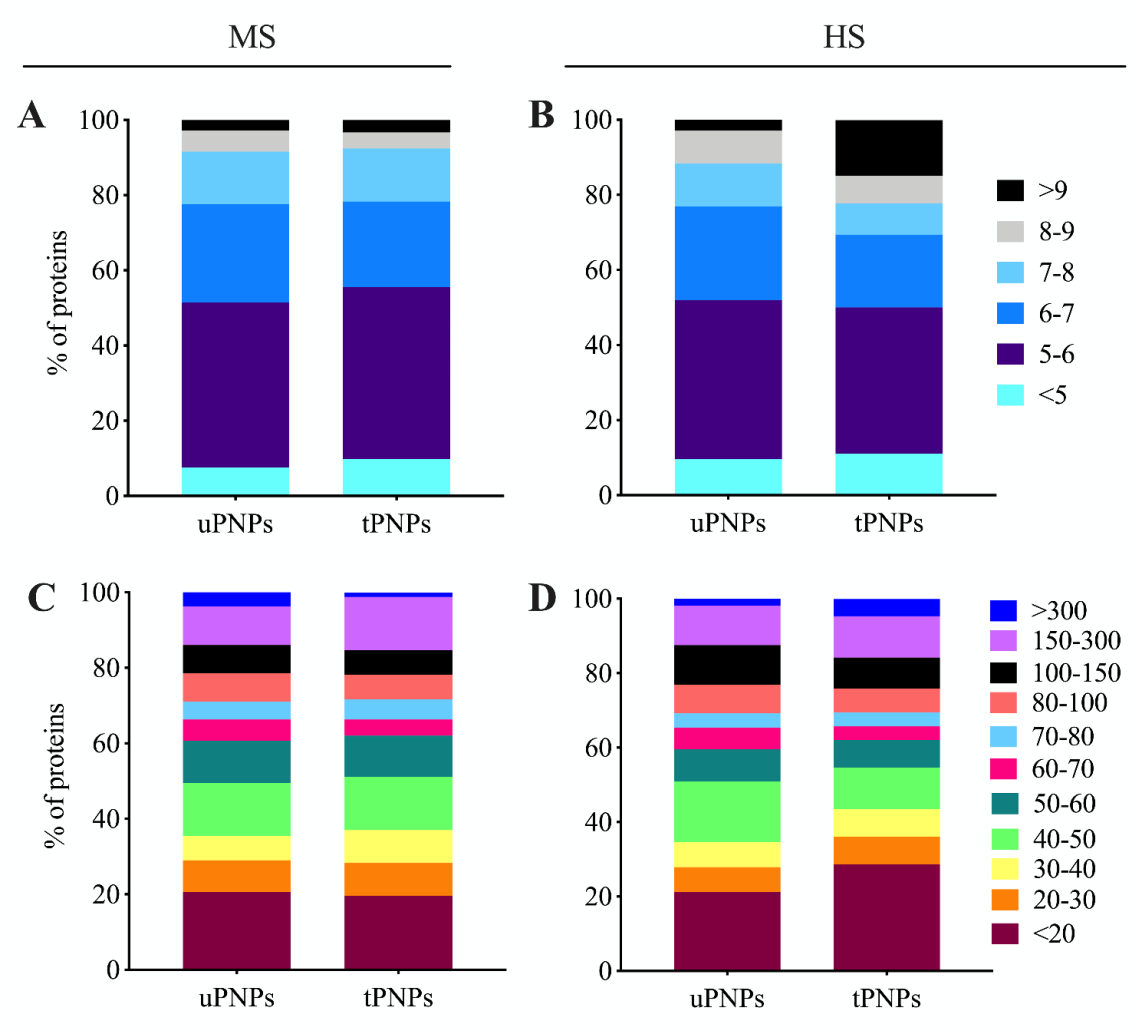
Classification of adsorbed proteins based on molecular weight and isoelectric point. Mouse and human proteins adsorbed to PNPs were classified based on their molecular weight (**A, B**) and isoelectric point (**C, D**). The graphs were plotted with the following proportion: total number of proteins detected : 100% = sum of proteins with the same caharacteristics : x. MS: mouse serum; HS: human serum; uPNPs: untargeted nanoparticles; tPNPs: targeted nanoparticles

The classification of proteins adsorbed on PNPs and based on MW and isoelectric point (IP) is part of their physical characterization. Both aspects can be used to predict the biochemical functions of the detected molecules [26]. This analysis also allows comparison between different species, such as those considered in the study: Homo Sapiens and Mus Musculus. In both HPC and MPC, proteins with a negative charge in serum predominate. Indeed, MPC of both PNPs was mainly represented by proteins with an IP between < 7, which accounted for 78% of the total proteins detected (Fig. 2A). In contrast, when the PNPs were incubated with HS, the differences were evident. Negatively charged proteins (IP < 7) accounted for 77% of the HPC of the uPNPs, while this percentage decreased for the tPNPs (69%). Another significant difference between particles is in human proteins with an IP > 9; such proteins, which carry a positive charge at physiological pH, were detected three times more frequently on tPNPs, where they accounted for up to 15% of the HPC, than on the untargeted counterpart (3%, Fig. 2B).

IP analysis represents the distribution of proteins based on their charge and allows predictions about the possible electrostatic interactions between PNPs and proteins. If serum proteins bound to PNPs only by electrostatic interactions, the adsorption pattern observed would be easily predictable. Due to the negative charge of the particles used in this study [25], we had expected to detect a greater number of positively-charged proteins in the PC. However, it is known that the surface charge of PNPs affects the conformational changes of proteins [27], which prevents their interaction with PNPs based on their charge alone. Furthermore, the PNPs used in the study consist of hydrophobic polymers such as PLA and PCL, and hydrophilic PEG. Hydrophobic polymers are known to bind mainly negatively charged molecules [28]. In our settings, the PCs of both PNPs were characterized by negatively charged proteins rather than proteins carrying a positive charge.

PNPs were also shown to preferentially adsorb small proteins. About 50% of the MPC consisted of proteins with MW < 50 kDa (uPNPs vs. tPNPs: 50% vs. 51%), of which 20% consisted of proteins smaller than 20 kDa. The only difference between the particles is in proteins with high MW (MW between 150 kDa and 300 kDa), which account for 10% and 14% of the MPC of uPNPs and tPNPs, respectively. This pattern is opposite taking in consideration proteins with MW above 300 kDa (uPNPs vs. tPNPs: 4% vs. 1%; Fig. 2C). When incubated with HS, about 50% of the proteins are those with MW below 50 kDa (uPNPs vs. tPNPs: 51% vs. 55%), as shown in mice. Among them, PNPs are mainly characterized by proteins with less than 20 kDa; they accounted for 20% and 29% of the HPC of uPNPs and tPNPs, respectively. Another difference between the particles is in human proteins between 40 and 50 kDa, which were adsorbed more by uPNPs than by tPNPs (uPNPs vs. tPNPs: 16% vs. 11%; Fig. 2D).

### Human protein corona differentiated more uPNPs from tPNPs in respect to the murine counterpart

**Fig. 3:**
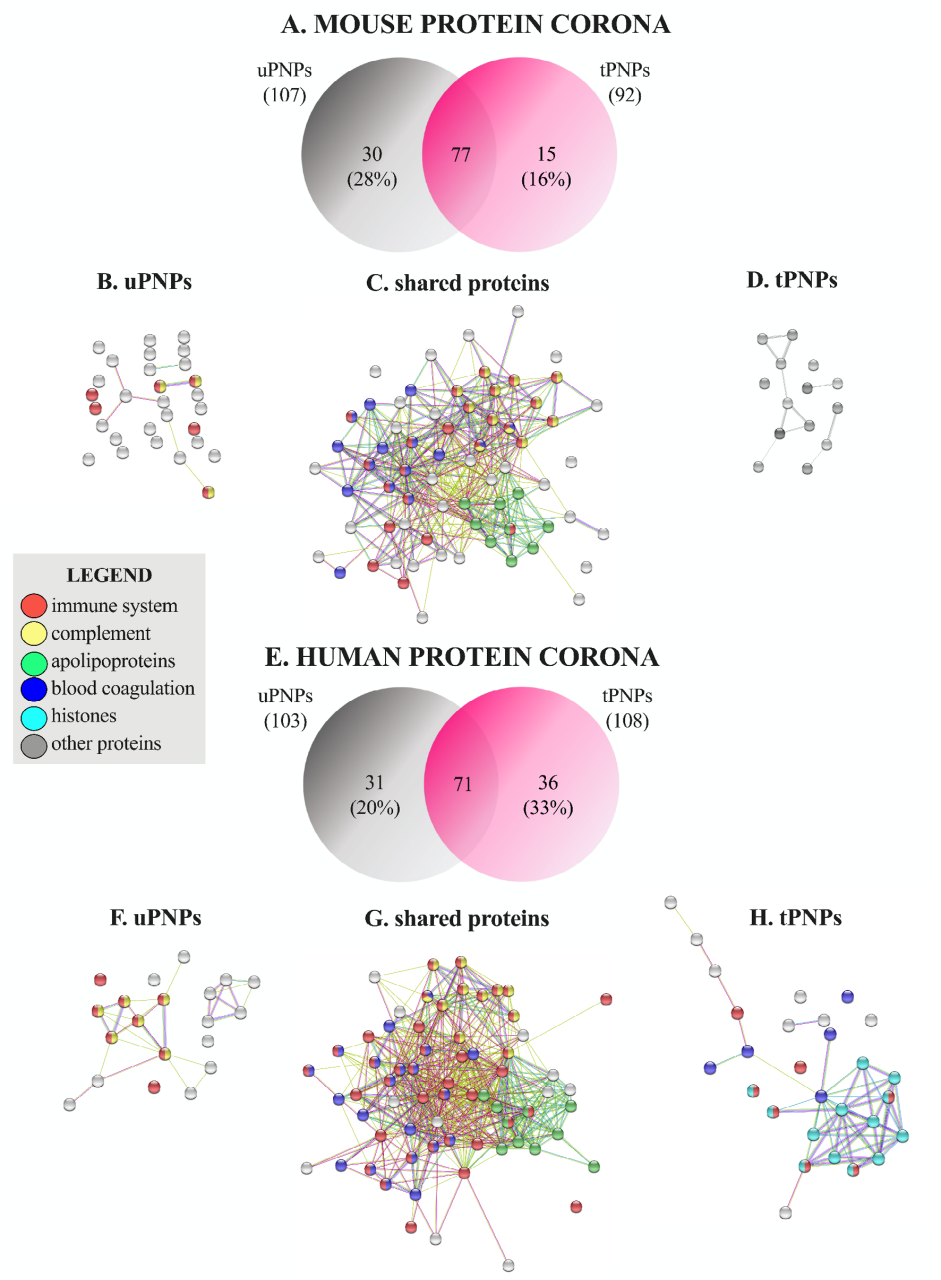
Venn diagram and biological function of adsorbed murine and human proteins. The proteins adsorbed to the PNPs were detected by LC-MS/MS and classified according to their biological function (e.g., immune system involvement, complement activation, blood coagulation, apolipoproteins, histones, and others). The number and percentage of mouse proteins are shown in a Venn diagram (**A**). Proteins adsorbed only on uPNPs (**B**), those shared by the particles (**C**) and those present only on tPNPs (**D**) are shown by string analysis. The same was for human proteins: Venn diagram (**E**), proteins adsorbed to uPNPs (**F**) or shared by both particles (**G**) or present only on tPNPs (**H**). uPNPs: untargeted nanoparticles; tPNPs: targeted nanoparticles

A more detailed classification of MPC and HPC was carried out, considering singularly the bound proteins. The Venn diagram showed the presence of 77 common murine proteins along with additional 30 unique proteins (28%) adsorbed to uPNPs, which is twice the proteins detected in the MPC of tPNPs (15 proteins, 16%, Fig. 3A).

The difference between PNPs is due to some proteins involved in C activation (Fig. 3B), including C1r and C1s, subcomponents of C1, and C9 (Additional File 2: Supplementary Table 1), and involved in immune response (Fig. 3B), such as antileukoproteinase, Beta-2-microglobulin and histidine-rich glycoprotein (Additional File 2: Supplementary Table 1). In addition to these proteins, apolipoproteins (Apos) are of great importance in a PC, as they play a significant role in blood circulation, in PNPs capacity to cross biological barriers [26] and in C activation as well. As for the late process, Apo A-I and Apo A-II inhibit C9 polymerization and hinder the formation of the membrane attack complex (MAC) [29]. A similar role is played by Apo J (also known as clusterin), which, in synergy with vitronectin, inactivates the terminal complexes C5b-9 [30]. All detected Apos (Apo A-I, Apo A-II, Apo A-IV, Apo B-100, Apo C-I, Apo C-III, Apo C-IV, Apo E and Apo M; Additional File 2: Supplementary Table 1) were shared by both particle types (Fig. 3C); Apo B-100 and Apo A-IV represent the 4th and the 5th most abundant proteins of the MPC of uPNPs (Additional File 2: Supplementary Table 1). The tPNPs did not adsorb unique proteins involved in the immune system, C activation or coagulation processes (Fig. 3D). This pattern was partially consistent with that found after incubating the particles with HS. Indeed, 72 common proteins were shared by both PNPs, and 31 (30%) and 36 (33%) additional unique proteins were detected on the surface of uPNPs and tPNPs, respectively (Fig. 3E). The differences between PNPs were primarily due to proteins involved in C activation, the coagulation process, and histones. A slightly higher number of human proteins involved in the C-activation pathway, such as C4, C6, C8 (beta and gamma chains), factor B and factor H (Additional File 3: Supplementary Table 2), were detected only on the surface of uPNPs (Fig. 3F). On the other hand, the HPC of tPNPs was more enriched in proteins involved in the coagulation process, including coagulation factor X and XIII-A, integrin α-Ib and ß-3, and multimerin-1 (Additional File 3: Supplementary Table 2).

As for human Apos, Apo A-I, Apo A-II, Apo A-IV, Apo B-100, Apo C-I, Apo C-II, Apo C-III, Apo C-IV, Apo D and Apo E (Additional File 3: Supplementary Table 2) were present on both PNPs (Fig. 3G); abundances were also comparable (data not shown). Apo B-100 and Apo E represent the 1st and 4th most abundant proteins of the HPC of both particles. Apo A-I is the 5th most abundant protein adsorbed on tPNPs (Additional File 3: Supplementary Table 2). Surprisingly, a higher number of histones, mainly H2B, was detected in the HPC of tPNPs (Fig. 3H). The binding of histones to PNPs is nothing new in the literature. Indeed, negatively charged PEGylated liposomes [31] and colloidal gold PNPs [32] have been shown to bind core histones such as H2A, H2B, H4 and H2B, H4, respectively. Free histones are generally released after necrosis of dying cells and are part of neutrophil extracellular traps (NETs); in our context, their involvement in immune activation and repair processes [33] could influence the faith of PNPs after injection. However, their function is still unknown.

### Murine serum fails to clearly distinguish uPNPs from tPNPs

**Fig. 4:**
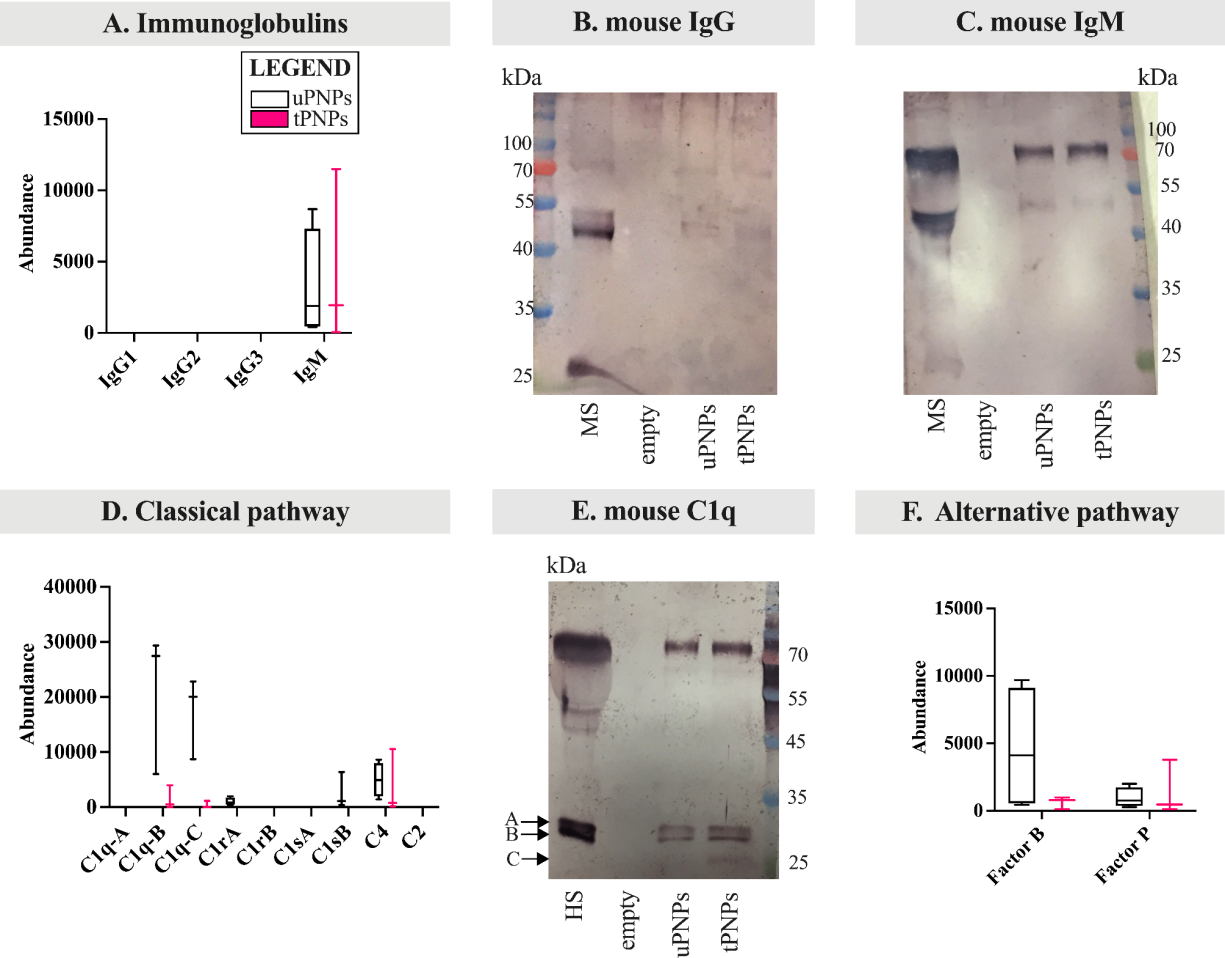
Deposition of components of the mouse complement system on PNPs. The proteins adsorbed on PNPs were analyzed by LC-MS/MS and Western blot (WB) analyses. Immunoglobulins were quantified by LC-MS/MS (**A**) and the deposition of mouse IgG (**B**) and IgM (**C**) was also analyzed by WB (attended band at 50 kDa and 75 kDa, respectively). Components of the classical complement activation pathway were analyzed by LC-MS/MS analysis (**D**); the corresponding bands of C1q chains were visualized by WB (**E**, attended bands height: ~26 and 27 kDa). Proteins of the alternative pathway (**F**) deposited on particles were detected by LC-MS/MS analysis. LC-MS/MS data (n=4) are presented as abundance values (arbitrary values generated by mass spec peak integration) and are shown as boxplot with median value. uPNPs: untargeted nanoparticles; tPNPs: targeted nanoparticles; MS: mouse serum; empty: empty well

With more attention to C deposition and/or activation, we have focused on the different components of the cascade, along with immunoglobulins (Igs). Regarding the latter, murine IgG (e.g., IgG2 and IgG3) and IgM isotypes are known to strongly activate murine C [34]. Among them, IgG were not adsorbed to PNPs, while IgM were more present in the MPC of tPNPs compared to uPNPs (Fig. 4A). These data, obtained by LC-MS/MS analysis, were also confirmed by WB. The IgG band (mouse IgG Fc (fragment crystallizable) region: 50 kDa) was not visible (Fig. 4B), while the constant region of IgM was detected on both particles with the attended size of 75 kDa (Fig. 4C).

IgG and IgM are responsible for activating the classical pathway of C after the binding to the C1 complex [35], which in turn consists of three subcomponents essential for its activity: C1q, formed from three chains (A, B and C), C1r and C1s [36]. After activation, C1 cleaves C4 and C2 into larger (C4b, C2b) and smaller (C4a, C2a) fragments. The catalytic activity of C1q leads to the formation of the C4bC2b complex, known as “C3 convertase”, which subsequently cleaves C3 into the anaphylatoxin C3a and the opsonin C3b [37]. Absorbed components of the classical C activation pathway by both PNPs include the B and C chains of C1q, C1r, C1s and C4. Except for C4, the other proteins were more abundant on uPNPs, which was also the only type of nanostructure that bound C1rA and C1sB. The latter proteins are one of the two isoforms resulting from gene duplication in mice that leads to the formation of C1rA, C1rB and C1sA, C1sB [38]. C1rB and C1sA were not detected (Fig. 4D). The deposition of C1q was also confirmed by WB analysis; the two bands of murine C1q (A chain (UNIPROT P98086 - C1QA_MOUSE) and C chain (UNIPROT Q02105 - C1QC_MOUSE): 25.974 kDa and 25.992 kDa, respectively, and B chain (UNIPROT P14106 - C1QB_MOUSE): 26.717 kDa) were visible on both particles (Fig. 4E). The band at ~ 75 kDa is the result of aspecific binding of the secondary antibody recognizing also IgM-Fc portion.

To further distinguish between C deposition and activation, the products of C4 cleavage were analyzed separately. C4a, a potent anaphylatoxin, is released from the C4 molecule to stimulate inflammation, while C4b remains on the cell surface to recruit other C components and continue the activation cascade [37]. Thus, when C is activated, C4a should not be detected. To assess the presence of this fragment, LC-MS/MS peptides were mapped to the original sequence of C4a, indicating that it is, at least partially, adsorbed on both particles and not a specific binding consequence of C1 activation (Additional File 4: Supplementary Fig. 2A).

Alternative pathway is initiated by the spontaneous auto-activation of C3 (C3H_2_O) into two molecules: C3a and C3b. C3b binds the target and then Factor B, cleaved by Factor D, to form C3bBb; this complex is further stabilized by Factor P [9]. In our settings, Factor B and Factor P were detected on both particles, but with opposite binding patterns: Factor B was more strongly adsorbed by uPNPs, while tPNPs showed higher abundance of Factor P (Fig. 4F).

Proteins specifically involved in the lectin pathway were not detected (data not shown).

**Fig. 5:**
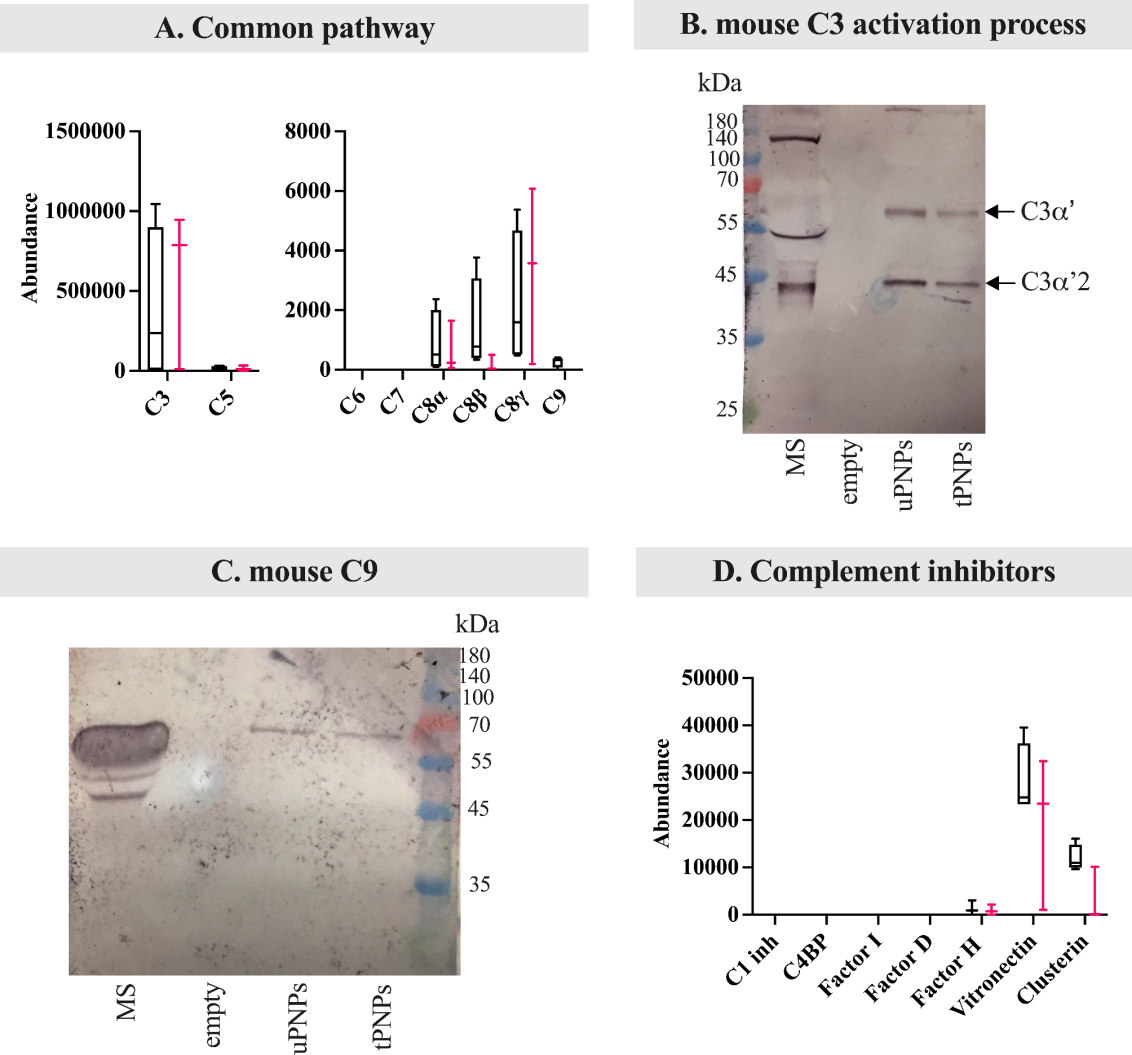
Deposition of mouse common complement components and inhibitors of the complement system on PNPs. The proteins adsorbed on PNPs were analyzed by LC-MS/MS (data presented as abundance values, arbitrary values generated by mass spec peak integration, n = 4) and Western blot (WB) analyses. LC-MS/MS data are shown as boxplot with median value. The deposition of common complement proteins (e.g., C3, C5, C6, C7, C8, C9) was quantified by LC-MS/MS analyses (**A**). To better evaluate complement activation, C3 fragmentation was studied by WB (**B**, C3α’: 63 kDa; C3α’2: 40 kDa). WB was also used to highlight the deposition of C9 (**C**, attended band at ~ 75 kDa), a terminal component of the complement activation. Complement inhibitors (**D**) were quantified by LC-MS/MS analysis. uPNPs: untargeted nanoparticles; tPNPs: targeted nanoparticles; MS: mouse serum; C1 inh: C1 inhibitor; C4BP: C4 binding proteins; empty: empty well

C3 acts as a convergence point of the three C pathways and was shown to be present on both PNPs, at the same abundance (Fig. 5A).

In addition, the detection of C3a, usually released from the C3 molecule after C activation [37], may provide an indication of its status. Peptides mapped in the C3a region were present on both uPNPs and tPNPs, suggesting only a partial C3 activation (Additional File 4: Supplementary Fig. 2B), as previously described for C4. This hypothesis was supported by the presence of C3 activation bands in the WB analysis. In addition to the loss of C3a (9 kDa) from the C3α chain of C3, there is also its fragmentation into smaller parts: C3α’ (63 kDa), C3α’2 (40 kDa) and C3f (2 kDa). C3α’ is cleaved again into two parts: one of 23 kDa and C3dg (40 kDa); the C3β chain remains intact (75 kDa) [39] (Additional File 4: Supplementary Fig. 2C). The WB analysis showed only two other visible bands at ~ 60 kDa and ~ 40 kDa, representing C3α’ and C3α’2, respectively. The band corresponding to the small fragment of C3α’ (23 kDa) was not detected (Fig. 5B).

The classical and alternative pathways converge to the same final steps: another C3 molecule binds to the previously formed C3 convertase to form C5 convertases (C4bC2bC3b and C3bBbC3b as C5 convertases of the classical alternative pathway, respectively), which in turn cleaves C5 to generate C5a and C5b. Specifically, C5a is released from the molecule, while C5b forms the MAC through the association with C6, C7, C8 and C9 and lyses cells [37]. Looking more closely at the late pathway, only C5 and C8 (α, ß and γ chains) were detected on the surface of both PNPs, while C9 was only evidenced on uPNPs. As with C4a and C3a, peptides mapping to the sequence of C5a were also analyzed: C5a was not present to the PNPs (Additional File 4: Supplementary Fig. 2D), indicating the complete activation of bound C5. C6 and C7 were not detected (Fig. 5A). The WB analysis also confirmed the presence of C9 to uPNPs; however, the attended band at ~ 75 kDa [40] was also visible on tPNPs (Fig. 5C).

To avoid constitutive activation of C in inappropriate contexts, there are several inhibitory proteins that act at different stages of the pathway. Briefly, the classical C activation pathway is inhibited by C1 inhibitor and C4BP. C1 inhibitor binds to the active site of C1r and C1s and inhibits the activated C1 complex [41], while C4BP controls the assembly of C3 convertase. C4BP is also involved in the regulation of the lectin pathway which causes the cleavage and inactivation of C4b. Through its multiple binding sites for C3b, Factor H also regulates the alternative C pathway and blocks the binding of factor B to form C3 convertase. Other inhibitory proteins that affect all three pathways are Factor I, Factor D, vitronectin and clusterin. Specifically, Factor I, along with cofactors such as Factor H and C4BP, cleaves C3b and C4b [42]. Factor D counteracts the formation of C3 convertase; specifically, for the alternative pathway, Factor D cleaves Factor B when it is bound to C3b [43]. Finally, clusterin and vitronectin associate with terminal complexes (C5b-9, C5b-7, C5b-8) to prevent their insertion into lipid bilayers of membranes [44]. After incubation with MS, only Factor H, clusterin, and vitronectin were adsorbed in almost equal amounts on both PNPs, whereas C1 inhibitor, C4BP, Factor D, and Factor I were not detected (Fig. 5D). All detected C inhibitors finally interfere with the deposition of the late components and the formation of MAC, so that their detection on both PNPs indicates that murine C activation can be prevented by this mechanism. Except for C1 complex components, which were more adsorbed on uPNPs, there are little differences between particles suggesting that mouse serum is unable to differentiate targeted from untargeted nanostructures in vitro.

### The antibody conjugated to tPNPs mediates the preferential binding of proteins of the human classical pathway, while the polymers preferentially bind molecules of the human alternative pathway

**Fig. 6:**
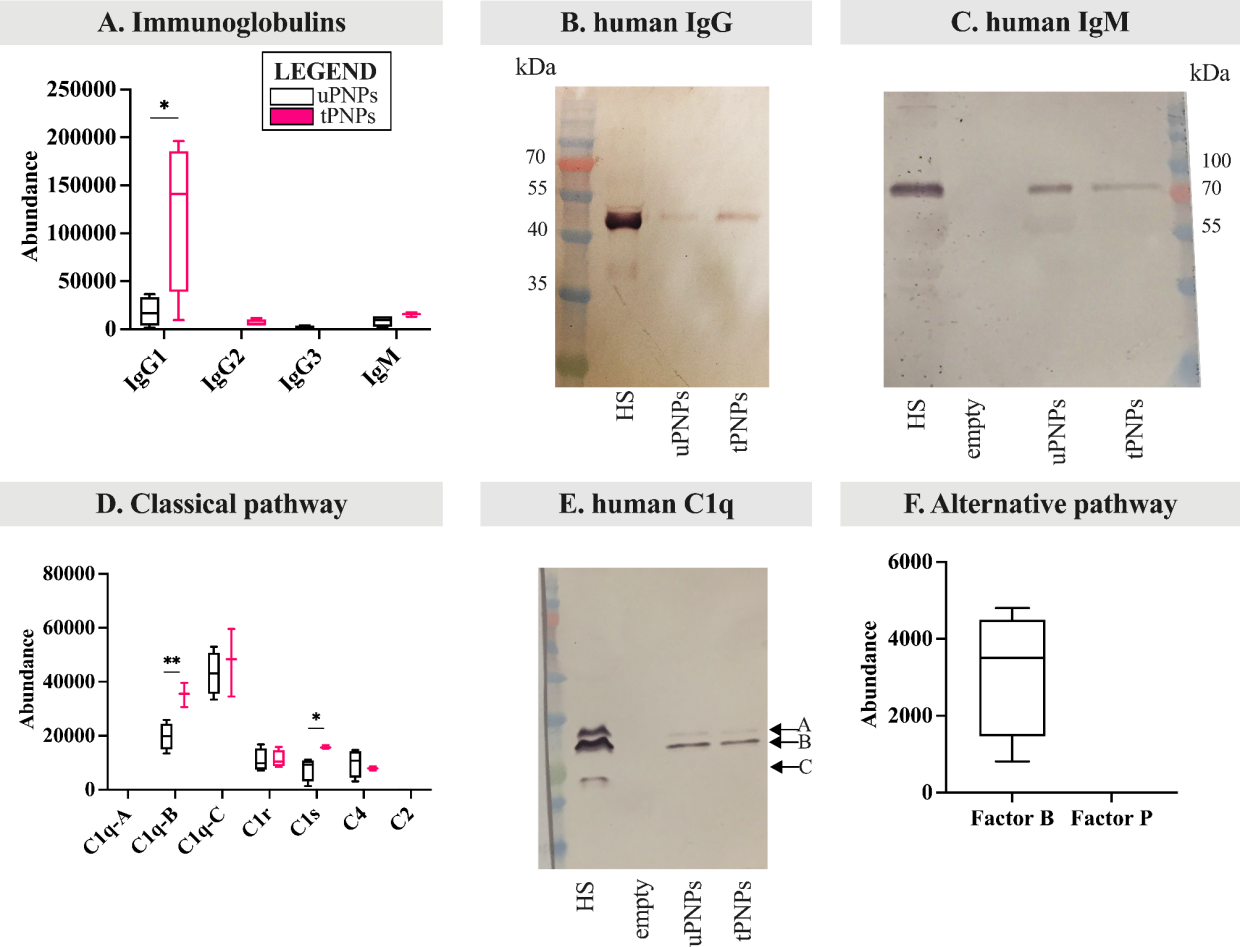
Deposition of proteins of the human complement system on PNPs. The adsorbed proteins were analyzed by LC-MS/MS and Western blot (WB) analyses. **A.** Adsorbed immunoglobulins such as IgG (i.e., IgG1, IgG2, IgG3) and IgM were detected by LC-MS/MS. The deposition of human IgG (**B**) and IgM (**C**) was also analyzed by WB analysis (attended band: 50 kDa and 75 kDa, respectively). Components of the classical complement activation pathway were analyzed by LC-MS/MS analysis (**D**) and the bands corresponding to C1q chains were visualized by WB (**E**, attended bands of C1q at ~ 23, 27 and 29 kDa). Proteins of the alternative (**F**) pathway were detected by LC-MS/MS analysis. LC-MS/MS data are shown as boxplots with median value. * p-value < 0.05; ** p-value < 0.01. uPNPs: untargeted nanoparticles; tPNPs: targeted nanoparticles; HS: human serum; HP: human plasma; empty: empty well

The same approach used for MPC, to assess the deposition of C system proteins, was also used for HPC. As for Igs, IgG1, IgG2, IgG3 and IgM are known to strongly activate the human C system [45, 46]. Therefore, our attention was initially focused on the adsorption of these molecules. tPNPs were shown to present greater amounts of IgG1 (uPNPs vs. tPNPs: 17,895 ± 15,421 vs. 121,895 ± 80,129, p-value 0.029) and low amount of IgG2, which was absent on uPNPs, and IgM; on the other hand, uPNPs bind low amount IgG3, which is absent in the HPC of tPNPs (Fig. 6A).

IgG adsorption was confirmed by WB analysis (human IgG Fc portion, 50 kDa; Fig. 6B). The very high abundance of human IgG on tPNPs with respect to uPNPs can be explained by the presence of the human-Fc antibody conjugated to the surface, which does not necessarily indicate a specific binding pattern of IgG to tPNPs but a control of the approach we were using. As for IgM, the band corresponding to the Fc portion (75 kDa, Fig. 6C) was more intense when uPNPs were considered, which is in contradiction with the LC-MS/MS data (Fig. 6A).

The classical C activation pathway is mediated by the C1 complex, as described for mice. The higher abundance of IgG1 and IgM detected on tPNPs corresponds to increased deposition of the C1 complex. Even though chain A of C1q was not detected and chain C was present on both particles without significant difference, chain B of C1q was significantly more documented on tPNPs than on uPNPs (uPNPs vs. tPNPs: 19,773 ± 5064 vs. 35,266 ± 4529, p-value 0.009). The same pattern was observed for C1s, which was statistically more abundant on tPNPs than on their untargeted counterpart (uPNPs vs. tPNPs: 7766 ± 4390 vs. 15,806 ± 630, p-value 0.033). On the other hand, C1r was adsorbed onto both particles in a similar abundance (Fig. 6D). The deposition of C1q was also investigated by WB analysis. The presence of the three visible bands through which C1q is formed (C1q chain A, B and C: 29, 27 and 23 kDa, respectively [47]) confirmed its adsorption on both PNPs (Fig. 6E). To assess the activation of C via the classical pathway, C2 and C4 molecules were also considered. C2 was undetected, while C4, including C4a (Additional File 5: Supplementary Fig. 3A), was adsorbed by both particles (Fig. 6D). These data suggested partial or lack of C activation.

**Fig. 7:**
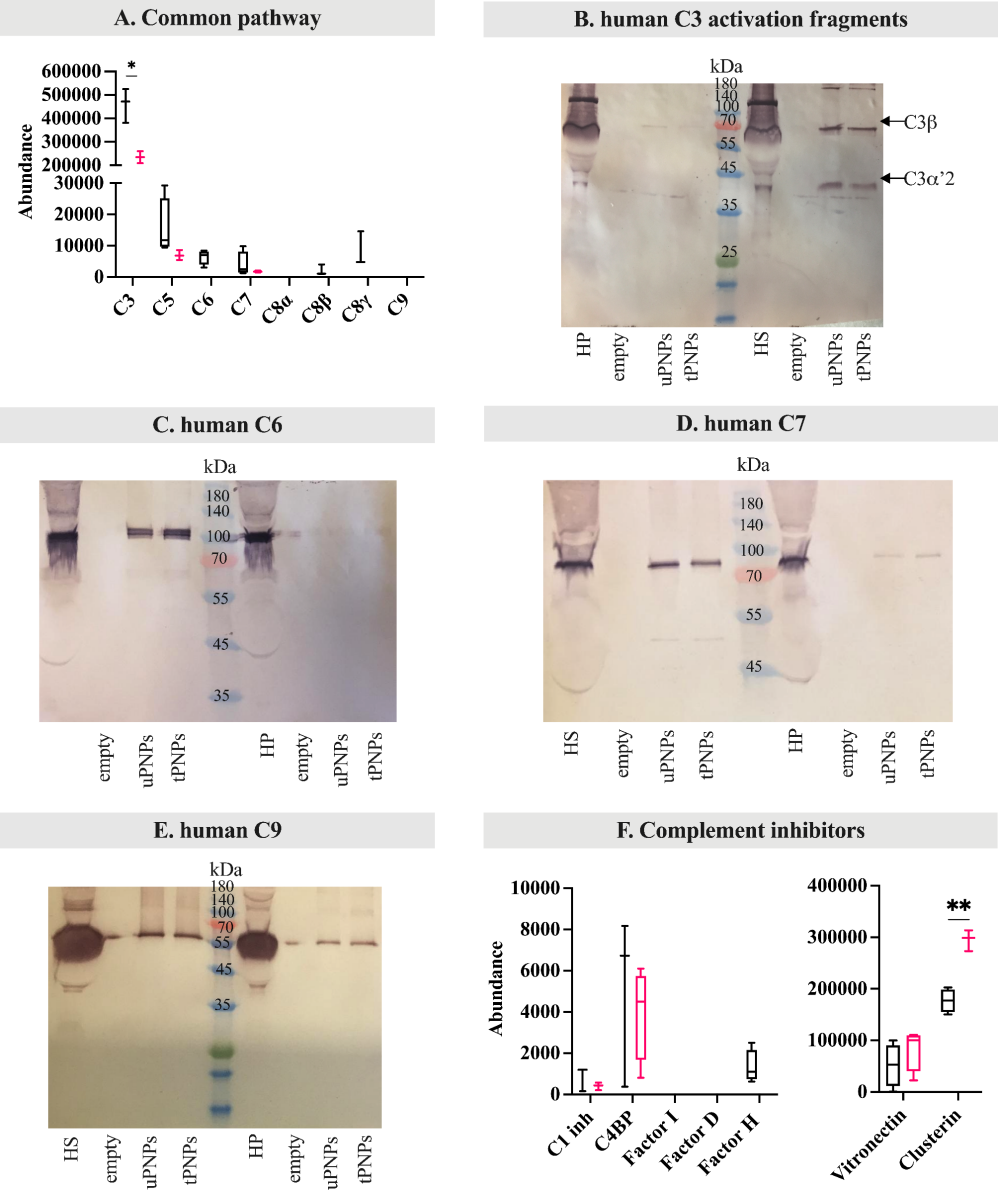
Deposition of human common pathway complement proteins and inhibitors on PNPs. The adsorbed proteins were analyzed by LC-MS/MS and Western blot (WB) analyses. The deposition of proteins of the common pathway of complement activation (C3, C5, C6, C7, C8 and C9) was quantified by LC-MS/MS (**A**). To better evaluate complement activation, C3 fragmentation was analyzed by WB (**B**, C3ß: 63 kDa; C3α’2: 40 kDa). WB was also used to visualize the deposition of C6 (**C**, attended band at ~ 100 kDa), C7 (**D**, attended band at ~ 95 kDa) and C9 (**E**, attended band at ~ 70 kDa). Complement inhibitors were quantified by LC-MS/MS analysis (**F**). uPNPs: untargeted nanoparticles; tPNPs: targeted nanoparticles; HS: human serum; HP: human plasma; empty: empty well; C1 inh: C1 inhibitor; C4BP: C4 binding protein

The alternative pathway of C activation in humans follows the same steps previously described for mice. In our settings, Factor B was documented only on uPNPs, while it was absent on tPNPs. Factor P was undetected at all (Fig. 6F). Along with Factor B, C3 also showed significantly higher abundance on uPNPs compared to tPNPs (uPNPs vs. tPNPs: 459,079 ± 72,796 vs. 234,834 ± 22,377, p-value 0.001; Fig. 7A), confirming the correlation between these two molecules during the activation of the alternative pathway; indeed, spontaneous auto-activation of soluble C3 or reaction of C3b with hydroxyl or amino groups of PNPs is known to mediate the formation of C3bBb convertase.

This, in turn, leads to an enhancement of C3b opsonization and thus activation of the alternative pathway [9].

The differences between untargeted and targeted PNPs are evident: tPNPs preferentially adsorbed components of the classical C activation pathway in accordance with the presence of the human-Fc antibody, while uPNPs were more prone to adsorb proteins of the alternative pathway.

To assess C activation, mapping of LC-MS/MS peptides to C3a and WB analysis of C3 fragments were performed. C3a was present on both NP systems, again indicating partial or absence of C activation (Additional File 5: Supplementary Fig. 3B). This result was in line with the WB analysis; after incubation with HS, two bands were visible: one at ~ 75 kDa and one at ~ 40 kDa corresponding to C3β and C3α’2 (or C3dg), respectively. The absence of other bands, such as those at 63 kDa (C3α) and 23 kDa (C3α without C3dg fragment), as well as the absence of significant bands after the incubation with HP (where the activation of the C system was blocked by the presence of EDTA), suggests C activation at least up to C3 level (Fig. 7B).

The components of the late pathway, in particular C5, C6 and C8 (ß and γ chains), were predominantly adsorbed on uPNPs. On the contrary, C7 was present in the HPC of both particles, while C9 was undetected (Fig. 7A). As already studied for C3 and in mouse, peptides corresponding to the sequence of C5a were detected only in the HPC of uPNPs, while they were absent on tPNPs (Additional File 5: Supplementary Fig. 3C). The absence of C5a on the HPC of tPNPs suggested the complete activation by the C5 convertases; probably, just a quote of C3 and C4 were hydrolyzed, due to the C3a and C4a detection on tPNPs.

An additional WB analysis was performed to assess the binding of C6, C7 and C9, comparing the incubation of the PNPs with HS or HP. C6 and C7 were detected on both particles in the presence of HS; indeed, the attended bands were visible at ~ 100 and ~ 95 kDa [48], respectively. With plasma, on the other hand, C6 and C7 were not detected, suggesting an activation at least up to C7 level (Fig. 7C and D, respectively). A discrepancy arose when C9 was considered. In fact, C9 was not present after LC-MS/MS analysis, whereas the attended band at ~ 70 kDa [48] was visible in WB after incubation of the PNPs with HS but also with HP, suggesting a binding independent from C activation (Fig. 7E).

As for inhibitors, C1 inhibitor, C4, vitronectin and clusterin were detected on both PNPs, while Factor H was adsorbed only on the surface of the uPNPs. Among them, clusterin showed significantly increased abundance on tPNPs (uPNPs vs. tPNPs: 176,928 ± 22,377 vs. 295,173 ± 20,315, p-value 0.001; Fig. 7F). The presence of alternative and late pathway components and the absence of inhibitors on uPNPs suggests possible activation of C from the beginning to the end by the alternative pathway. In contrast, the absence of late pathway proteins and the presence of a significantly higher amount of inhibitors adsorbed on tPNPs indicated less C activation on these particles.

### tPNPs activated more C through classical and lectin pathways

**Fig. 8:**
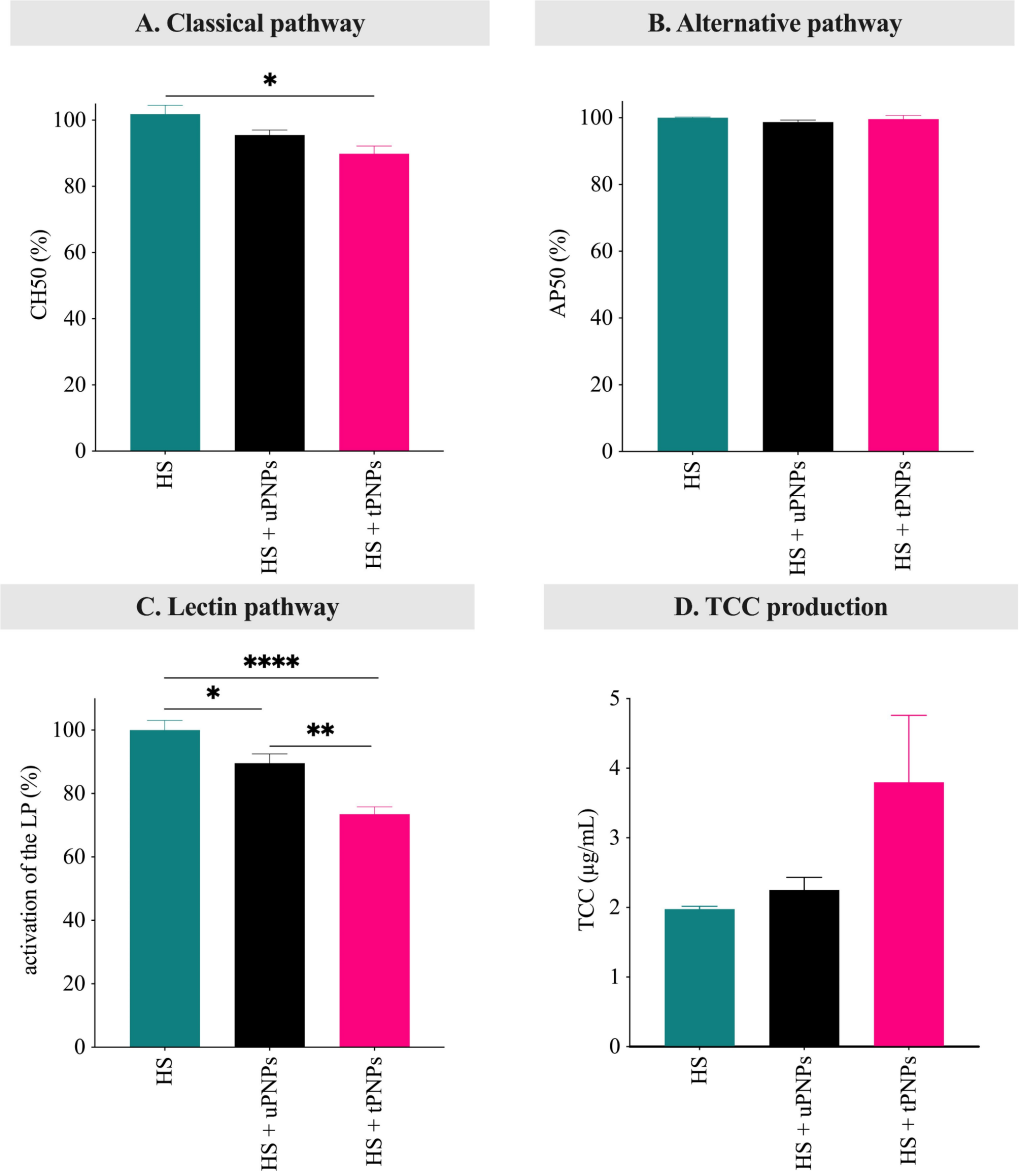
Activation of the human complement by PNPs. Classical (**A**) and alternative (**B**) pathway activation was evaluated by lytic assays. The activation of lectin pathway (**C**) and the deposition of TCC (**D**) were assessed by ELISA. Data are shown as mean ± SEM, n = 3. Data were analyzed by one-way ANOVA. * p-value < 0.05; ** p-value < 0.01; *** p-value < 0.001; **** p-value < 0.0001. HS: human serum; uPNPs: untargeted nanoparticles; tPNPs: targeted nanoparticles; CH50: 50% of hemolytic activity induced by the classical pathway; AP50: 50% of hemolytic activity induced by the alternative pathway; LP: lectin pathway; abs: absorbance; TCC: terminal complement complex

LC-MS/MS analysis, together with WB, provides indications of C activation status. To fully elucidate the contribution of the targeting mechanism conjugated to the PNPs in C activation, hemolytic assays and ELISA were performed to evaluate residual C activity and C-activation products in human serum. Hemolytic assays have traditionally been based on the simple photometric measurement of hemoglobin release, following lysis of RBCs mediated by C activation. This method can selectively exploit classical or alternative C activation pathways using Ab-sensitized sheep RBCs [49] or rabbit RBCs [50]. Although mouse often mimics human processes, HS remains the preferred sample for C activation studies in vitro. Indeed, it has been demonstrated that in vitro hemolytic activity of mouse C is approximately 200- to 300-fold lower than human C [51]. PNPs were incubated with HS and, after centrifugation, the supernatant containing the residual C activity was incubated with sheep or rabbit RBCs. Untreated HS was used as a positive control, retaining the total amount of C activity. Consistent with the higher deposition of C1 complex components on tPNPs, they were shown to slightly reduce the residual activity of the classical pathway (Fig. 8A; HS vs. tPNPs-treated HS: 100 ± 4.2 vs. 89.9 ± 4; p-value 0.034).

Two further discrepancies were found when examining activation of the alternative and lectin pathway. Although Factor B, C3 and the late pathway proteins adsorbed more to uPNPs, no significant activation of the alternative pathway was detected for any of the nanostructures (Fig. 8B). Furthermore, no deposition of lectin pathway proteins was detected by LC-MS/MS. Surprisingly, the lectin pathway was the most reduced among the three pathways. In fact, both PNPs were found to significantly activate the lectin pathway (HS vs. uPNPs-treated HS: 100 ± 7.4 vs. 89.6 ± 7.1, p-value 0.044. HS vs. tPNPs-treated HS: 100 ± 7.4 vs. 73.5 ± 5.6, p-value < 0.0001), with tPNPs causing significantly higher C consumption compared to uPNPs (uPNPs vs. tPNPs: 89.6 ± 7.1 vs. 73.5 ± 5.6, p-value 0.0025. Fig. 8C). This can be due to a reduction of the concentration of other components shared with the different C pathway; in particular, C4 and C2 with the classical pathway and C3 and the late components with both classical and alternative pathway.

To confirm C activation, the production of the TCC was evaluated detecting C5b-C9 complex by ELISA. Both particles showed TCC production after incubation with HS but without significant difference between particles (Fig. 8D).

### The activation of C on PNPs affects macrophages engulfment but is not influenced by targeting abs

**Fig. 9:**
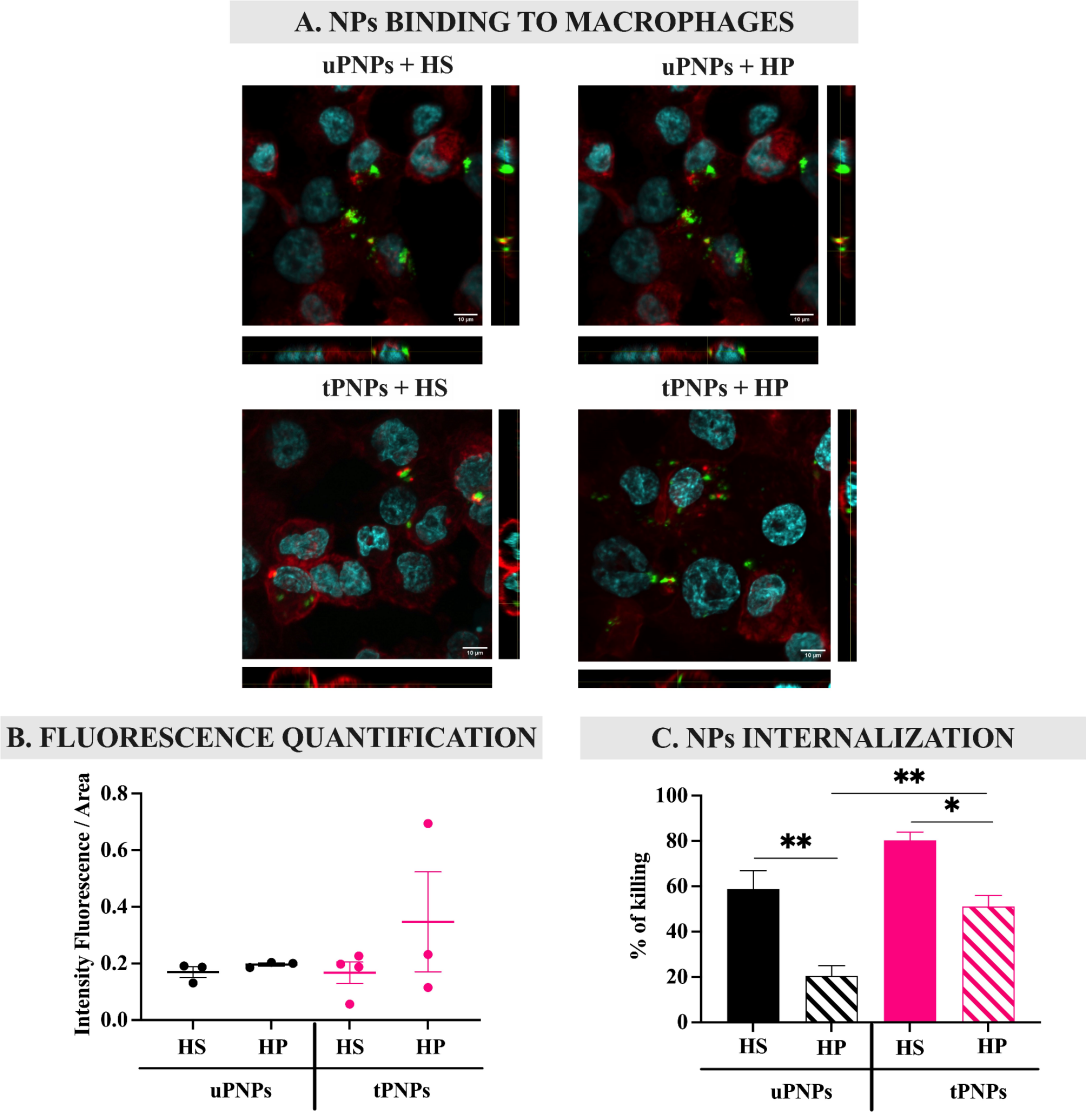
PNPs binding and internalization inside macrophages after complement activation. PMA-stimulated THP-1 cells were incubated with HS- or HP-PNPs for 1 h. Confocal microscopy images were acquired (**A**) and the amount of fluorescence was quantified with Fiji software (**B**). To assess PNPs internalization inside cells, drugs-loaded PNPs were incubated for 1 h with human macrophages. Unbound PNPs were removed from cells and the percentage of dying cells was quantified by MTT assay (**C**). Data are represented as mean ± SEM; n = 3. Statistical analysis was performed using a one-way ANOVA test. * p-value < 0.05; ** p-value < 0.01. uPNPs: untargeted nanoparticles; tPNPs: targeted nanoparticles; HS: human serum; HP: human plasma

The liver is the main site of elimination of PNPs after injection [23]. This process is mediated by the large number of macrophages in this organ, which, in turn, express on their surface specific receptors for foreign structures [7] that have been opsonized by molecules, including C activated products. Therefore, the contribution of C in the interaction of nanostructures with macrophages was assessed in vitro by comparing PNPs incubated with HS (causing C deposition) and HP (in which the C system is blocked). In brief, fluorescent PNPs were incubated with HS or HP and brought in contact with human macrophages (PMA-stimulated THP-1 cells); the binding of the PNPs to the cells was analyzed by confocal microscopy (Fig. 9A).

Macrophages were found to interact with PNPs independently of C activation; indeed, incubation of PNPs with HS did not increase binding to the cells compared with HP (Fig. 9B). However, the difference between the particles became clear evaluating PNPs internalization,  which was assessed by quantifying the percentage of macrophages killed after exposure to drugs-loaded PNPs. First, tPNPs without C activation (tPNPs + HP) were internalized significantly more than the untargeted counterpart (uPNPs vs. tPNPs: 20.4 ± 9.1% vs. 51.2 ± 9.6%, p-value 0.004). This pattern is probably due to the binding of the human Fc portion of the antibody conjugated to tPNPs to specific receptors expressed on macrophages, including THP-1 cells [52]. After incubation with HS, the internalization of both PNPs in the cells increased significantly compared to HP (uPNPs HP vs. HS: 20.4 ± 9.1% vs. 58.8 ± 13.9%, p-value 0.021. tPNPs HP vs. HS: 51.2 ± 9.6% vs. 80.3 ± 6.2%, p-value 0.005), confirming the link between C activation and macrophage engulfment, but no differences were observed comparing targeted and untargeted particles incubated with HS (Fig. 9C); these data demonstrate a similar opsonization of the nanostructure, not sufficient to increase macrophage engulfment, independent from the presence of the targeting antibody and inducing a similar final elimination by phagocytosis after PC formation.

## Conclusion

This approach based on LC-MS/MS allows to deeply study PC. We applied the method to compare mouse and human PC on PNPs, aiming to further see the results of surface modification with PEG or with antibodies as targeting agents. The C system is fundamental in the elimination of NPs after injection into living organisms. Thus, we focused on the deposition and activation of the C system on NPs. The results clearly evidence the presence of C proteins on PNPs surface but is important to underlie that part of them derived from an unspecific deposition rather than an effective C activation. The presence of a targeting antibody favors the activation of the classical pathway, but its absence does not prevent activation of C. In fact, uPNPs were demonstrated to increase the activation of the alternative pathway resulting in similar opsonization of PNPs and tPNPs and similar phagocytosis by macrophages.

It also remains evident the importance of comparing biochemical and function results. Even if these particles showed the presence of C activation products on their surface, clearly indicating a possible elimination mechanism, the adsorption of C inhibitors limits the activation of the system, ultimately not impairing the activity of circulating C system and, as a result, not enhancing the susceptivity to infection that is typical of C-deficient patients.

### Electronic supplementary material

Below is the link to the electronic supplementary material.

Supplementary Material 1

Supplementary Material 2

Supplementary Material 3

Supplementary Material 4

Supplementary Material 5

Supplementary Material 6

## Data Availability

All data are available in the main text or the supplementary materials.
