# Supplementary material for: Surface antibody changes protein corona both in human and mouse serum but not final opsonization and elimination of targeted polymeric nanoparticles
Source: J Nanobiotechnology. 2023 Oct 14;21:376. doi: 10.1186/s12951-023-02134-4 (PMC10576379; doi:10.1186/s12951-023-02134-4)
Supplement: Supplementary file 1 — Supplementary Material 1 [file 12951_2023_2134_MOESM1_ESM.docx]

| uPNPs | SHARED | tPNPs |
| --- | --- | --- |
| COAGULATION | | |
|  | Antithrombin-III |  |
|  | Band 3 anion transport protein |  |
|  | Carboxypeptidase B2 |  |
|  | Coagulation factor V (u3, t4) |  |
|  | Coagulation factor X |  |
|  | Coagulation factor XIII A chain |  |
|  | Complement C3 (u1, t1) |  |
|  | Complement factor H |  |
|  | Fibrinogen alpha chain (t5) |  |
|  | Fibrinogen beta chain |  |
|  | Fibrinogen gamma chain |  |
|  | Glia-derived nexin |  |
|  | Kininogen-1 |  |
|  | Platelet factor 4 |  |
|  | Prothrombin |  |
| COMPLEMENT | | |
| Complement C1r-A subcomponent | Complement C1q subcomponent subunit B |  |
| Complement C1s-B subcomponent | Complement C1q subcomponent subunit C |  |
| Complement component C9 | Complement C1s-A subcomponent |  |
|  | Complement C3 (u1, t1) |  |
|  | Complement C4-B |  |
|  | Complement C5 |  |
|  | Complement component C8 alpha chain |  |
|  | Complement component C8 beta chain |  |
|  | Complement component C8 gamma chain |  |
|  | Complement factor B |  |
|  | Complement factor H |  |
|  | Properdin |  |
| IMMUNE SYSTEM | | |
| Antileukoproteinase | 14-3-3 protein zeta/delta |  |
| Beta-2-microglobulin | Actin, cytoplasmic 2 |  |
| Complement C1r-A subcomponent | Antithrombin-III |  |
| Complement C1s-B subcomponent | Apolipoprotein A-IV (u5) |  |
| Complement component C9 | Complement C1q subcomponent subunit B |  |
| Histidine-rich glycoprotein | Complement C1q subcomponent subunit C |  |
|  | Complement C1s-A subcomponent |  |
|  | Complement C3 (u1, t1) |  |
|  | Complement C4-B |  |
|  | Complement C5 |  |
|  | Complement component C8 alpha chain |  |
|  | Complement component C8 beta chain |  |
|  | Complement component C8 gamma chain |  |
|  | Complement factor B |  |
|  | Complement factor H |  |
|  | Fibrinogen alpha chain (t5) |  |
|  | Fibrinogen beta chain |  |
|  | Fibrinogen gamma chain |  |
|  | Gelsolin |  |
|  | Glyceraldehyde-3-phosphate dehydrogenase |  |
|  | Platelet factor 4 |  |
|  | Properdin |  |
|  | Prothrombin |  |
|  | Vitronectin |  |
| APOLIPOPROTEINS | | |
|  | Apolipoprotein A-I |  |
|  | Apolipoprotein A-II |  |
|  | Apolipoprotein A-IV (u5) |  |
|  | Apolipoprotein B-100 (u4) |  |
|  | Apolipoprotein C-I |  |
|  | Apolipoprotein C-III |  |
|  | Apolipoprotein C-IV |  |
|  | Apolipoprotein E |  |
|  | Apolipoprotein M |  |
| OTHERS | | |
| Actin, alpha cardiac muscle 1 | Actin, cytoplasmic 1 | Alpha-2-antiplasmin |
| ADP-ribosylation factor 5 | Alpha-1-antitrypsin 1-2 | Ankyrin-1 |
| Alpha-1-antitrypsin 1-1 | Alpha-1-antitrypsin 1-3 | Beta-actin-like protein 2 |
| Angiopoietin-1 | Alpha-1-antitrypsin 1-5 | Cathelicidin antimicrobial peptide |
| Caspase recruitment domain-containing protein 10 | Alpha-2-HS-glycoprotein | Fermitin family homolog 3 |
| Ceruloplasmin | BPI fold-containing family A member 2 | Hemopexin |
| Cytoplasmic dynein 2 heavy chain 1 | Carboxylesterase 1C | Ig heavy chain V region 3 |
| Dynein heavy chain 2, axonemal | Carboxypeptidase N catalytic chain | Integrin alpha-IIb |
| EH domain-containing protein 4 | Clusterin | Integrin beta-3 |
| Elongation factor 1-alpha 1 | Fibronectin (u2, t2) | Pleckstrin |
| Histone H4 | Filamin-A | Serum amyloid A-4 protein |
| Ig heavy chain V-III region A4 | Glutathione peroxidase 3 | Serum paraoxonase/arylesterase 1 |
| Insulin-like growth factor-binding protein 5 | H-2 class I histocompatibility antigen, Q10 alpha chain | Spectrin alpha chain, erythrocytic 1 |
| Inter-alpha-trypsin inhibitor heavy chain H1 | Hemoglobin subunit alpha | Spectrin beta chain, erythrocytic |
| Matrix metalloproteinase-19 | Hemoglobin subunit beta-1 | Vitamin D-binding protein |
| Neutrophilic granule protein | Hemoglobin subunit beta-2 |  |
| Phospholipid transfer protein | Ig gamma-2A chain C region secreted form |  |
| Profilin-1 | Ig heavy chain V-III region ABE-47N |  |
| Serine protease inhibitor A3K | Ig heavy chain V-III region E109 |  |
| Serum amyloid A-1 protein | Ig heavy chain V-III region J606 |  |
| Sulfhydryl oxidase 1 | Ig heavy chain V-III region T957 |  |
| Telomerase protein component 1 | Ig heavy chain V-III region U61 |  |
| Titin | Ig heavy chain V-III region W3082 |  |
| Tubulin polyglutamylase TTLL11 | Immunoglobulin heavy constant mu |  |
|  | Immunoglobulin kappa constant |  |
|  | Inter alpha-trypsin inhibitor, heavy chain 4 |  |
|  | Inter-alpha-trypsin inhibitor heavy chain H2 |  |
|  | Metalloproteinase inhibitor 3 |  |
|  | Murinoglobulin-1 |  |
|  | Murinoglobulin-2 |  |
|  | Phospholipase A1 member A |  |
|  | Pregnancy zone protein (t3) |  |
|  | Protein 4.1 |  |
|  | Serine protease inhibitor A3M |  |
|  | Serotransferrin |  |
|  | Serum albumin |  |
|  | Talin-1 |  |
|  | Thrombospondin-1 |  |
