# Supplementary figures and images for: Surface antibody changes protein corona both in human and mouse serum but not final opsonization and elimination of targeted polymeric nanoparticles
Source: J Nanobiotechnology. 2023 Oct 14;21:376. doi: 10.1186/s12951-023-02134-4 (PMC10576379; doi:10.1186/s12951-023-02134-4)

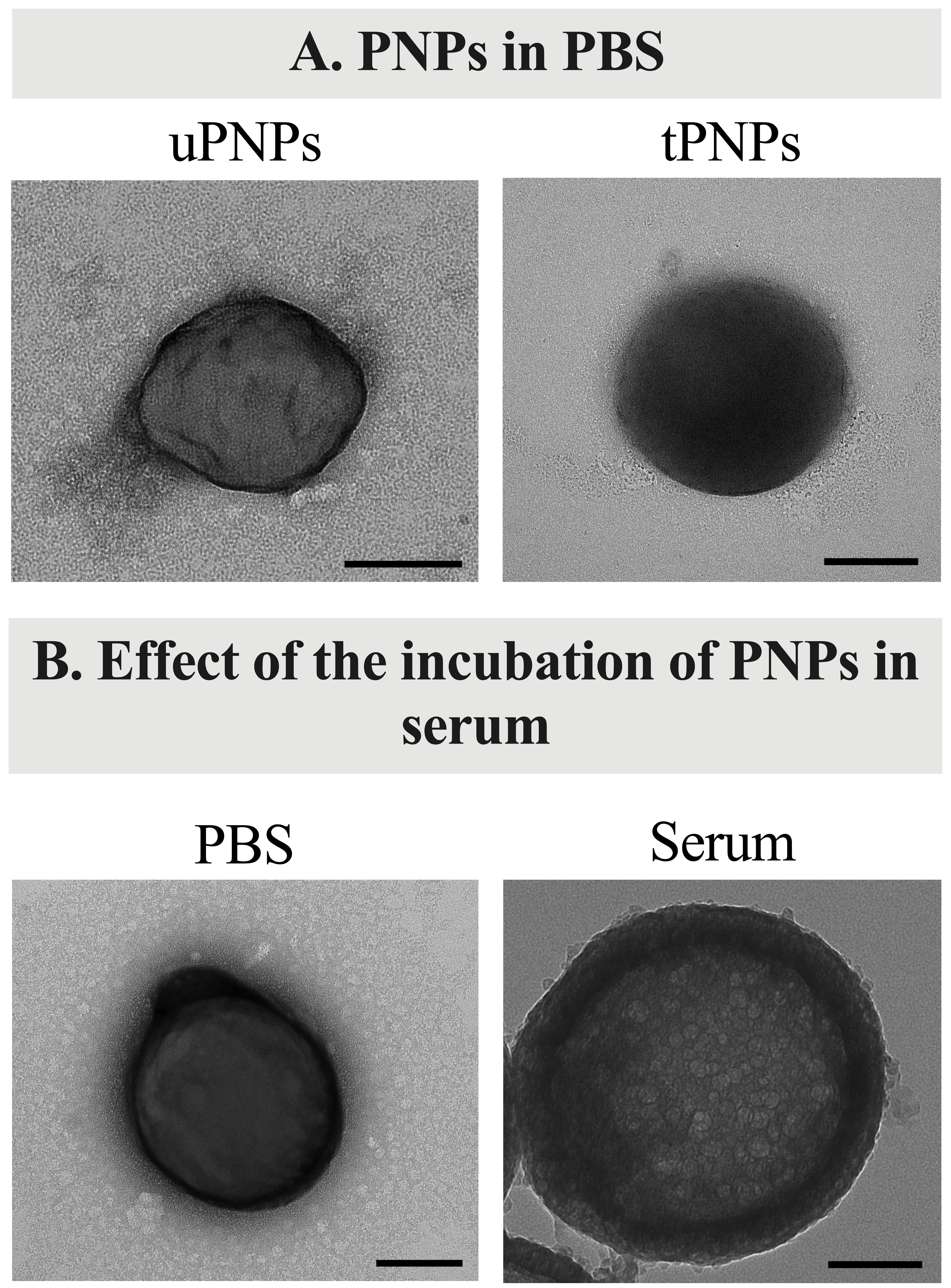

Supplement: Supplementary file 2 — Supplementary Material 2 [file 12951_2023_2134_MOESM2_ESM.png]
