# Supplementary material for: Surface antibody changes protein corona both in human and mouse serum but not final opsonization and elimination of targeted polymeric nanoparticles
Source: J Nanobiotechnology. 2023 Oct 14;21:376. doi: 10.1186/s12951-023-02134-4 (PMC10576379; doi:10.1186/s12951-023-02134-4)
Supplement: Supplementary file 3 — Supplementary Material 3 [file 12951_2023_2134_MOESM3_ESM.docx]

| uPNPs | SHARED | tPNPs | | |  |
| --- | --- | --- | --- | --- | --- |
| IMMUNE SYSTEM | | | | |  |
| Apolipoprotein L1 | Actin, cytoplasmic 1 | Complement C4-B | | |  |
| Complement C4-A | Actin, cytoplasmic 2 | Histone H2B type 1-C/E/F/G/I | | |  |
| Complement component C6 | Alpha-1-antichymotrypsin | Histone H2B type 1-J | | |  |
| Complement component C8 beta chain | Alpha-1-antitrypsin | Histone H2B type 1-K | | |  |
| Complement component C8 gamma chain | Alpha-2-HS-glycoprotein | Histone H2B type 2-E | | |  |
| Complement factor B | Antithrombin-III | Histone H2B type F-S | | |  |
| Complement factor H | Apolipoprotein A-IV | Immunoglobulin J chain | | |  |
| N-acetylmuramoyl-L-alanine amidase | Apolipoprotein B-100 (u1, t1) |  | | |  |
|  | Band 3 anion transport protein |  | | |  |
|  | C4b-binding protein alpha chain |  | | |  |
|  | Cathepsin G |  | | |  |
|  | Clusterin |  | | |  |
|  | Complement C1q subcomponent subunit B |  | | |  |
|  | Complement C1q subcomponent subunit C |  | | |  |
|  | Complement C1r subcomponent |  | | |  |
|  | Complement C1s subcomponent |  | | |  |
|  | Complement C3 (u2, t2) |  | | |  |
|  | Complement C5 |  | | |  |
|  | Complement component C7 |  | | |  |
|  | Complement component C9 |  | | |  |
|  | Fibrinogen alpha chain |  | | |  |
|  | Fibrinogen beta chain |  | | |  |
|  | Fibronectin (u3, t3) |  | | |  |
|  | Filamin-A |  | | |  |
|  | Gelsolin |  | | |  |
|  | Glyceraldehyde-3-phosphate dehydrogenase |  | | |  |
|  | Haptoglobin |  | | |  |
|  | Hemoglobin subunit beta |  | | |  |
|  | Heparanase |  | | |  |
|  | Hornerin | |  | | |
|  | Kininogen-1 | |  | | |
|  | Plasma protease C1 inhibitor | |  | | |
|  | Plasminogen | |  | | |
|  | Platelet factor 4 | |  | | |
|  | Proteoglycan 4 | |  | | |
|  | Prothrombin | |  | | |
|  | Serotransferrin | |  | | |
|  | Thrombospondin-1 | |  | | |
|  | Transthyretin | |  | | |
|  | Vitronectin | |  | | |
| COMPLEMENT | | | | |  |
| Complement C4-A | C4b-binding protein alpha chain | Complement C4-B | | |  |
| Complement component C6 | Clusterin |  | | |  |
| Complement component C8 beta chain | Complement C1q subcomponent subunit B |  | | |  |
| Complement component C8 gamma chain | Complement C1q subcomponent subunit C |  | | |  |
| Complement factor B | Complement C1r subcomponent |  | | |  |
| Complement factor H | Complement C1s subcomponent |  | | |  |
|  | Complement C3 (u2, t2) |  | | |  |
|  | Complement C5 |  | | |  |
|  | Complement component C7 |  | | |  |
|  | Complement component C9 |  | | |  |
|  | Plasma protease C1 inhibitor |  | | |  |
| APOLIPOPROTEINS | | | | |  |
|  | Apolipoprotein A-I (t5) |  | | |  |
|  | Apolipoprotein A-II |  | | |  |
|  | Apolipoprotein A-IV |  | | |  |
|  | Apolipoprotein B-100 (u1, t1) |  | | |  |
|  | Apolipoprotein C-I |  | | |  |
|  | Apolipoprotein C-II |  | | |  |
|  | Apolipoprotein C-III |  | | |  |
|  | Apolipoprotein C-IV |  | | |  |
|  | Apolipoprotein D |  | | |  |
|  | Apolipoprotein E (u4, t4) |  | | |  |
|  | Apolipoprotein(a) |  | | |  |
| COAGULATION | | | | |  |
|  | 14-3-3 protein zeta/delta | Coagulation factor X | | |  |
|  | Actin, cytoplasmic 1 | Coagulation factor XIII A chain | | |  |
|  | Actin, cytoplasmic 2 | Integrin alpha-Ib | | |  |
|  | Alpha-1-antitrypsin | Integrin beta-3 | | |  |
|  | Alpha-2-macroglobulin (u5) | Multimerin-1 | | |  |
|  | Antithrombin-III |  | | |  |
|  | Band 3 anion transport protein |  | | |  |
|  | Carboxypeptidase B2 |  | | |  |
|  | Coagulation factor V |  | | |  |
|  | Coagulation factor VIII |  | | |  |
|  | Fibrinogen alpha chain |  | | |  |
|  | Fibrinogen beta chain |  | | |  |
|  | Fibrinogen gamma chain |  | | |  |
|  | Filamin-A |  | | |  |
|  | Hemoglobin subunit beta |  | | |  |
|  | Kininogen-1 |  | | |  |
|  | Plasma protease C1 inhibitor |  | | |  |
|  | Plasminogen |  | | |  |
|  | Platelet factor 4 |  | | |  |
|  | Prothrombin |  | | |  |
|  | Talin-1 |  | | |  |
| HISTONES | | | |  |  |
|  |  | Histone H2B type 1-B | | |  |
|  |  | Histone H2B type 1-C/E/F/G/I | | |  |
|  |  | Histone H2B type 1-D | | |  |
|  |  | Histone H2B type 1-H | | |  |
|  |  | Histone H2B type 1-J | | |  |
|  |  | Histone H2B type 1-K | | |  |
|  |  | Histone H2B type 1-L | | |  |
|  |  | Histone H2B type 1-M | | |  |
|  |  | Histone H2B type 1-N | | |  |
|  |  | Histone H2B type 1-O | | |  |
|  |  | Histone H2B type 2-E | | |  |
|  |  | Histone H2B type 2-F | | |  |
|  |  | Histone H2B type 3-B | | |  |
|  |  | Histone H2B type F-S | | |  |
| OTHERS | | | | |  |
| Actin, alpha cardiac muscle 1 | Beta-actin-like protein 2 | ADP-ribosylation factor 5 | | |  |
| Actin, alpha skeletal muscle | Hemoglobin subunit alpha | Alpha-1-acid glycoprotein 1 | | |  |
| Actin, aortic smooth muscle | Immunoglobulin alpha-2 heavy chain | Centromere-associated protein E | | |  |
| Actin, gamma-enteric smooth muscle | Immunoglobulin gamma-1 heavy chain | Dynein heavy chain 14, axonemal | | |  |
| Cadherin-1 | Immunoglobulin heavy constant alpha 1 | Immunoglobulin heavy constant gamma 2 | | |  |
| Ceruloplasmin | Immunoglobulin heavy constant mu | Immunoglobulin heavy constant gamma 4 | | |  |
| Hemopexin | Immunoglobulin kappa light chain | Immunoglobulin heavy variable 5-51 | | |  |
| Immunoglobulin heavy constant gamma 3 | Immunoglobulin kappa variable 1-16 | Immunoglobulin kappa variable 3-11 | | |  |
| Immunoglobulin kappa variable 1-17 | Inter-alpha-trypsin inhibitor heavy chain H1 | Immunoglobulin lambda variable 3-21 | | |  |
| Immunoglobulin kappa variable 1-27 | Inter-alpha-trypsin inhibitor heavy chain H2 | Immunoglobulin lambda variable 3-9 | | |  |
| Immunoglobulin kappa variable 1-33 | Inter-alpha-trypsin inhibitor heavy chain H4 | Myeloid cell nuclear differentiation antigen | | |  |
| Immunoglobulin kappa variable 1-39 | POTE ankyrin domain family member E | Serum amyloid A-2 protein | | |  |
| Immunoglobulin kappa variable 1D-16 | POTE ankyrin domain family member I | Spectrin beta chain, erythrocytic | | |  |
| Immunoglobulin kappa variable 1D-33 | Serum albumin | Titin | | |  |
| Immunoglobulin kappa variable 1D-39 | Serum amyloid A-4 protein | Vitamin D-binding protein | | |  |
| Immunoglobulin lambda constant 2 | Serum paraoxonase/arylesterase 1 |  | | |  |
| Immunoglobulin lambda constant 3 |  |  | | |  |
| Immunoglobulin lambda constant 6 |  |  | | |  |
| Microtubule-associated protein RP/EB family member 2 |  |  | | |  |
| Ras GTPase-activating protein 4 |  |  | | |  |
| Ras GTPase-activating protein 4B |  |  | | |  |
