# Supplementary material for: Surface antibody changes protein corona both in human and mouse serum but not final opsonization and elimination of targeted polymeric nanoparticles
Source: J Nanobiotechnology. 2023 Oct 14;21:376. doi: 10.1186/s12951-023-02134-4 (PMC10576379; doi:10.1186/s12951-023-02134-4)
Supplement: Supplementary file 6 — Supplementary Material 6 [file 12951_2023_2134_MOESM6_ESM.docx]

**Surface antibody changes protein corona both in human and mouse serum but not final opsonization and elimination of targeted polymeric nanoparticles**

Sara Capolla^1^, Federico Colombo^2,3^, Luca De Maso^3^, Prisca Mauro^3^, Paolo Bertoncin^3^, Thilo Kähne^4^, Alexander Engler^4^, Luis Núñez^5,6,7^, Ruben Spretz^5,7^, Gustavo Larsen ^5,7,8^, Michele Dal Bo^1^, Giuseppe Toffoli^1^, Paolo Macor^3*^.

**Affiliations**

^1^ Experimental and Clinical Pharmacology Unit, Centro di Riferimento Oncologico di Aviano (CRO), Istituto di Ricovero e Cura a Carattere Scientifico (IRCCS), 33081 Aviano, Italy

^2^ Institute for Molecular Systems Engineering and Advanced Materials (IMSEAM), Ruprecht-Karls-Universität Heidelberg, Im Neuenheimer Feld 225, 69120 Heidelberg, Germany

^3^ Department of Life Sciences, University of Trieste, 34127 Trieste, Italy

^4^ Institute of Exptl. Internal Medicine, Medical Faculty, Otto von Guericke University, 39120 Magdeburg, Germany

^5^ BioTarget Inc, Chicago, IL, USA

^6^ Concordia University, Natural Science Department, 7400 Augusta St, River Forest, 60305, IL, USA

^7^ LNK Chemsolutions LLC, Lincoln, Nebraska, USA

^8^ University of Nebraska Lincoln, Department of Chemical and Biochemical Engineering, Lincoln, NE, USA

*** Corresponding author:** Paolo Macor, Department of Life Sciences, University of Trieste, via Giorgieri, 5 34127 Trieste, Italy. E-mail: pmacor@units.it. Phone: +39 040 5588683. ORCID: 0000-0003-3079-4019.

**Supplementary Figures legends**

**Additional File 1: Supplementary Figure 1**

**
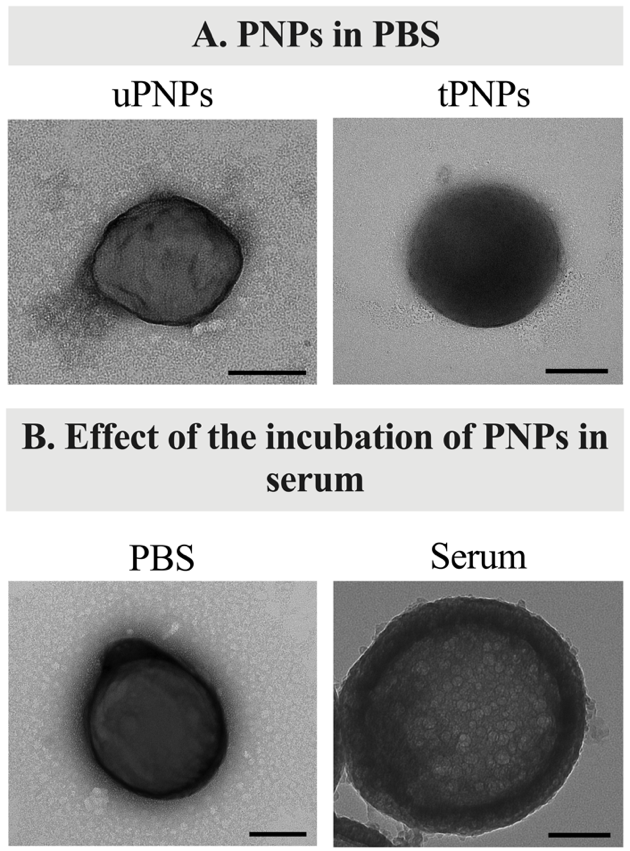
**

**Supplementary Figure 1. TEM images of the protein corona formation on PNPs.** Representative TEM image of uPNPs and tNPs before (**A**) and after (**B**) incubation with serum. PBS: phosphate buffered saline; PNPs: polymeric nanoparticles; uPNPs: untargeted PNPs; tPNPs: targeted PNPs. Scale bar: 100 nm.

**Additional File 2: Supplementary Table 1**

**Supplementary Table 1. List of murine proteins adsorbed on NPs.**

| uPNPs | SHARED | tPNPs |
| --- | --- | --- |
| COAGULATION | | |
|  | Antithrombin-III |  |
|  | Band 3 anion transport protein |  |
|  | Carboxypeptidase B2 |  |
|  | Coagulation factor V (u3, t4) |  |
|  | Coagulation factor X |  |
|  | Coagulation factor XIII A chain |  |
|  | Complement C3 (u1, t1) |  |
|  | Complement factor H |  |
|  | Fibrinogen alpha chain (t5) |  |
|  | Fibrinogen beta chain |  |
|  | Fibrinogen gamma chain |  |
|  | Glia-derived nexin |  |
|  | Kininogen-1 |  |
|  | Platelet factor 4 |  |
|  | Prothrombin |  |
| COMPLEMENT | | |
| Complement C1r-A subcomponent | Complement C1q subcomponent subunit B |  |
| Complement C1s-B subcomponent | Complement C1q subcomponent subunit C |  |
| Complement component C9 | Complement C1s-A subcomponent |  |
|  | Complement C3 (u1, t1) |  |
|  | Complement C4-B |  |
|  | Complement C5 |  |
|  | Complement component C8 alpha chain |  |
|  | Complement component C8 beta chain |  |
|  | Complement component C8 gamma chain |  |
|  | Complement factor B |  |
|  | Complement factor H |  |
|  | Properdin |  |
| IMMUNE SYSTEM | | |
| Antileukoproteinase | 14-3-3 protein zeta/delta |  |
| Beta-2-microglobulin | Actin, cytoplasmic 2 |  |
| Complement C1r-A subcomponent | Antithrombin-III |  |
| Complement C1s-B subcomponent | Apolipoprotein A-IV (u5) |  |
| Complement component C9 | Complement C1q subcomponent subunit B |  |
| Histidine-rich glycoprotein | Complement C1q subcomponent subunit C |  |
|  | Complement C1s-A subcomponent |  |
|  | Complement C3 (u1, t1) |  |
|  | Complement C4-B |  |
|  | Complement C5 |  |
|  | Complement component C8 alpha chain |  |
|  | Complement component C8 beta chain |  |
|  | Complement component C8 gamma chain |  |
|  | Complement factor B |  |
|  | Complement factor H |  |
|  | Fibrinogen alpha chain (t5) |  |
|  | Fibrinogen beta chain |  |
|  | Fibrinogen gamma chain |  |
|  | Gelsolin |  |
|  | Glyceraldehyde-3-phosphate dehydrogenase |  |
|  | Platelet factor 4 |  |
|  | Properdin |  |
|  | Prothrombin |  |
|  | Vitronectin |  |
| APOLIPOPROTEINS | | |
|  | Apolipoprotein A-I |  |
|  | Apolipoprotein A-II |  |
|  | Apolipoprotein A-IV (u5) |  |
|  | Apolipoprotein B-100 (u4) |  |
|  | Apolipoprotein C-I |  |
|  | Apolipoprotein C-III |  |
|  | Apolipoprotein C-IV |  |
|  | Apolipoprotein E |  |
|  | Apolipoprotein M |  |
| OTHERS | | |
| Actin, alpha cardiac muscle 1 | Actin, cytoplasmic 1 | Alpha-2-antiplasmin |
| ADP-ribosylation factor 5 | Alpha-1-antitrypsin 1-2 | Ankyrin-1 |
| Alpha-1-antitrypsin 1-1 | Alpha-1-antitrypsin 1-3 | Beta-actin-like protein 2 |
| Angiopoietin-1 | Alpha-1-antitrypsin 1-5 | Cathelicidin antimicrobial peptide |
| Caspase recruitment domain-containing protein 10 | Alpha-2-HS-glycoprotein | Fermitin family homolog 3 |
| Ceruloplasmin | BPI fold-containing family A member 2 | Hemopexin |
| Cytoplasmic dynein 2 heavy chain 1 | Carboxylesterase 1C | Ig heavy chain V region 3 |
| Dynein heavy chain 2, axonemal | Carboxypeptidase N catalytic chain | Integrin alpha-IIb |
| EH domain-containing protein 4 | Clusterin | Integrin beta-3 |
| Elongation factor 1-alpha 1 | Fibronectin (u2, t2) | Pleckstrin |
| Histone H4 | Filamin-A | Serum amyloid A-4 protein |
| Ig heavy chain V-III region A4 | Glutathione peroxidase 3 | Serum paraoxonase/arylesterase 1 |
| Insulin-like growth factor-binding protein 5 | H-2 class I histocompatibility antigen, Q10 alpha chain | Spectrin alpha chain, erythrocytic 1 |
| Inter-alpha-trypsin inhibitor heavy chain H1 | Hemoglobin subunit alpha | Spectrin beta chain, erythrocytic |
| Matrix metalloproteinase-19 | Hemoglobin subunit beta-1 | Vitamin D-binding protein |
| Neutrophilic granule protein | Hemoglobin subunit beta-2 |  |
| Phospholipid transfer protein | Ig gamma-2A chain C region secreted form |  |
| Profilin-1 | Ig heavy chain V-III region ABE-47N |  |
| Serine protease inhibitor A3K | Ig heavy chain V-III region E109 |  |
| Serum amyloid A-1 protein | Ig heavy chain V-III region J606 |  |
| Sulfhydryl oxidase 1 | Ig heavy chain V-III region T957 |  |
| Telomerase protein component 1 | Ig heavy chain V-III region U61 |  |
| Titin | Ig heavy chain V-III region W3082 |  |
| Tubulin polyglutamylase TTLL11 | Immunoglobulin heavy constant mu |  |
|  | Immunoglobulin kappa constant |  |
|  | Inter alpha-trypsin inhibitor, heavy chain 4 |  |
|  | Inter-alpha-trypsin inhibitor heavy chain H2 |  |
|  | Metalloproteinase inhibitor 3 |  |
|  | Murinoglobulin-1 |  |
|  | Murinoglobulin-2 |  |
|  | Phospholipase A1 member A |  |
|  | Pregnancy zone protein (t3) |  |
|  | Protein 4.1 |  |
|  | Serine protease inhibitor A3M |  |
|  | Serotransferrin |  |
|  | Serum albumin |  |
|  | Talin-1 |  |
|  | Thrombospondin-1 |  |

Light grey proteins are shared by more than one process. Numbers in parentheses indicate the 5 most abundant proteins (u: proteins adsorbed on uPNPs; t: proteins adsorbed on tPNPs). PNPs: polymeric nanoparticles; uPNPs: untargeted PNPs; tPNPs: targeted PNPs.

**Additional File 3: Supplementary Table 2**

**Supplementary Table 2. List of human proteins adsorbed on NPs**

| uPNPs | SHARED | tPNPs |
| --- | --- | --- |
| IMMUNE SYSTEM | | |
| Apolipoprotein L1 | Actin, cytoplasmic 1 | Complement C4-B |
| Complement C4-A | Actin, cytoplasmic 2 | Histone H2B type 1-C/E/F/G/I |
| Complement component C6 | Alpha-1-antichymotrypsin | Histone H2B type 1-J |
| Complement component C8 beta chain | Alpha-1-antitrypsin | Histone H2B type 1-K |
| Complement component C8 gamma chain | Alpha-2-HS-glycoprotein | Histone H2B type 2-E |
| Complement factor B | Antithrombin-III | Histone H2B type F-S |
| Complement factor H | Apolipoprotein A-IV | Immunoglobulin J chain |
| N-acetylmuramoyl-L-alanine amidase | Apolipoprotein B-100 (u1, t1) |  |
|  | Band 3 anion transport protein |  |
|  | C4b-binding protein alpha chain |  |
|  | Cathepsin G |  |
|  | Clusterin |  |
|  | Complement C1q subcomponent subunit B |  |
|  | Complement C1q subcomponent subunit C |  |
|  | Complement C1r subcomponent |  |
|  | Complement C1s subcomponent |  |
|  | Complement C3 (u2, t2) |  |
|  | Complement C5 |  |
|  | Complement component C7 |  |
|  | Complement component C9 |  |
|  | Fibrinogen alpha chain |  |
|  | Fibrinogen beta chain |  |
|  | Fibronectin (u3, t3) |  |
|  | Filamin-A |  |
|  | Gelsolin |  |
|  | Glyceraldehyde-3-phosphate dehydrogenase |  |
|  | Haptoglobin |  |
|  | Hemoglobin subunit beta |  |
|  | Heparanase |  |
|  | Hornerin |  |
|  | Kininogen-1 |  |
|  | Plasma protease C1 inhibitor |  |
|  | Plasminogen |  |
|  | Platelet factor 4 |  |
|  | Proteoglycan 4 |  |
|  | Prothrombin |  |
|  | Serotransferrin |  |
|  | Thrombospondin-1 |  |
|  | Transthyretin |  |
|  | Vitronectin |  |
| COMPLEMENT | | |
| Complement C4-A | C4b-binding protein alpha chain | Complement C4-B |
| Complement component C6 | Clusterin |  |
| Complement component C8 beta chain | Complement C1q subcomponent subunit B |  |
| Complement component C8 gamma chain | Complement C1q subcomponent subunit C |  |
| Complement factor B | Complement C1r subcomponent |  |
| Complement factor H | Complement C1s subcomponent |  |
|  | Complement C3 (u2, t2) |  |
|  | Complement C5 |  |
|  | Complement component C7 |  |
|  | Complement component C9 |  |
|  | Plasma protease C1 inhibitor |  |
| APOLIPOPROTEINS | | |
|  | Apolipoprotein A-I (t5) |  |
|  | Apolipoprotein A-II |  |
|  | Apolipoprotein A-IV |  |
|  | Apolipoprotein B-100 (u1, t1) |  |
|  | Apolipoprotein C-I |  |
|  | Apolipoprotein C-II |  |
|  | Apolipoprotein C-III |  |
|  | Apolipoprotein C-IV |  |
|  | Apolipoprotein D |  |
|  | Apolipoprotein E (u4, t4) |  |
|  | Apolipoprotein(a) |  |
| COAGULATION | | |
|  | 14-3-3 protein zeta/delta | Coagulation factor X |
|  | Actin, cytoplasmic 1 | Coagulation factor XIII A chain |
|  | Actin, cytoplasmic 2 | Integrin alpha-Ib |
|  | Alpha-1-antitrypsin | Integrin beta-3 |
|  | Alpha-2-macroglobulin (u5) | Multimerin-1 |
|  | Antithrombin-III |  |
|  | Band 3 anion transport protein |  |
|  | Carboxypeptidase B2 |  |
|  | Coagulation factor V |  |
|  | Coagulation factor VIII |  |
|  | Fibrinogen alpha chain |  |
|  | Fibrinogen beta chain |  |
|  | Fibrinogen gamma chain |  |
|  | Filamin-A |  |
|  | Hemoglobin subunit beta |  |
|  | Kininogen-1 |  |
|  | Plasma protease C1 inhibitor |  |
|  | Plasminogen |  |
|  | Platelet factor 4 |  |
|  | Prothrombin |  |
|  | Talin-1 |  |
| HISTONES | | |
|  |  | Histone H2B type 1-B |
|  |  | Histone H2B type 1-C/E/F/G/I |
|  |  | Histone H2B type 1-D |
|  |  | Histone H2B type 1-H |
|  |  | Histone H2B type 1-J |
|  |  | Histone H2B type 1-K |
|  |  | Histone H2B type 1-L |
|  |  | Histone H2B type 1-M |
|  |  | Histone H2B type 1-N |
|  |  | Histone H2B type 1-O |
|  |  | Histone H2B type 2-E |
|  |  | Histone H2B type 2-F |
|  |  | Histone H2B type 3-B |
|  |  | Histone H2B type F-S |
| OTHERS | | |
| Actin, alpha cardiac muscle 1 | Beta-actin-like protein 2 | ADP-ribosylation factor 5 |
| Actin, alpha skeletal muscle | Hemoglobin subunit alpha | Alpha-1-acid glycoprotein 1 |
| Actin, aortic smooth muscle | Immunoglobulin alpha-2 heavy chain | Centromere-associated protein E |
| Actin, gamma-enteric smooth muscle | Immunoglobulin gamma-1 heavy chain | Dynein heavy chain 14, axonemal |
| Cadherin-1 | Immunoglobulin heavy constant alpha 1 | Immunoglobulin heavy constant gamma 2 |
| Ceruloplasmin | Immunoglobulin heavy constant mu | Immunoglobulin heavy constant gamma 4 |
| Hemopexin | Immunoglobulin kappa light chain | Immunoglobulin heavy variable 5-51 |
| Immunoglobulin heavy constant gamma 3 | Immunoglobulin kappa variable 1-16 | Immunoglobulin kappa variable 3-11 |
| Immunoglobulin kappa variable 1-17 | Inter-alpha-trypsin inhibitor heavy chain H1 | Immunoglobulin lambda variable 3-21 |
| Immunoglobulin kappa variable 1-27 | Inter-alpha-trypsin inhibitor heavy chain H2 | Immunoglobulin lambda variable 3-9 |
| Immunoglobulin kappa variable 1-33 | Inter-alpha-trypsin inhibitor heavy chain H4 | Myeloid cell nuclear differentiation antigen |
| Immunoglobulin kappa variable 1-39 | POTE ankyrin domain family member E | Serum amyloid A-2 protein |
| Immunoglobulin kappa variable 1D-16 | POTE ankyrin domain family member I | Spectrin beta chain, erythrocytic |
| Immunoglobulin kappa variable 1D-33 | Serum albumin | Titin |
| Immunoglobulin kappa variable 1D-39 | Serum amyloid A-4 protein | Vitamin D-binding protein |
| Immunoglobulin lambda constant 2 | Serum paraoxonase/arylesterase 1 |  |
| Immunoglobulin lambda constant 3 |  |  |
| Immunoglobulin lambda constant 6 |  |  |
| Microtubule-associated protein RP/EB family member 2 |  |  |
| Ras GTPase-activating protein 4 |  |  |
| Ras GTPase-activating protein 4B |  |  |

Light grey proteins are shared by more than one process. Numbers in parentheses (1-5) indicate the 5 most abundant proteins (u: proteins adsorbed on uPNPs; t: proteins adsorbed on tPNPs). PNPs: polymeric nanoparticles; uPNPs: untargeted PNPs; tPNPs: targeted PNPs.

**Additional File 4: Supplementary Figure 2**

**
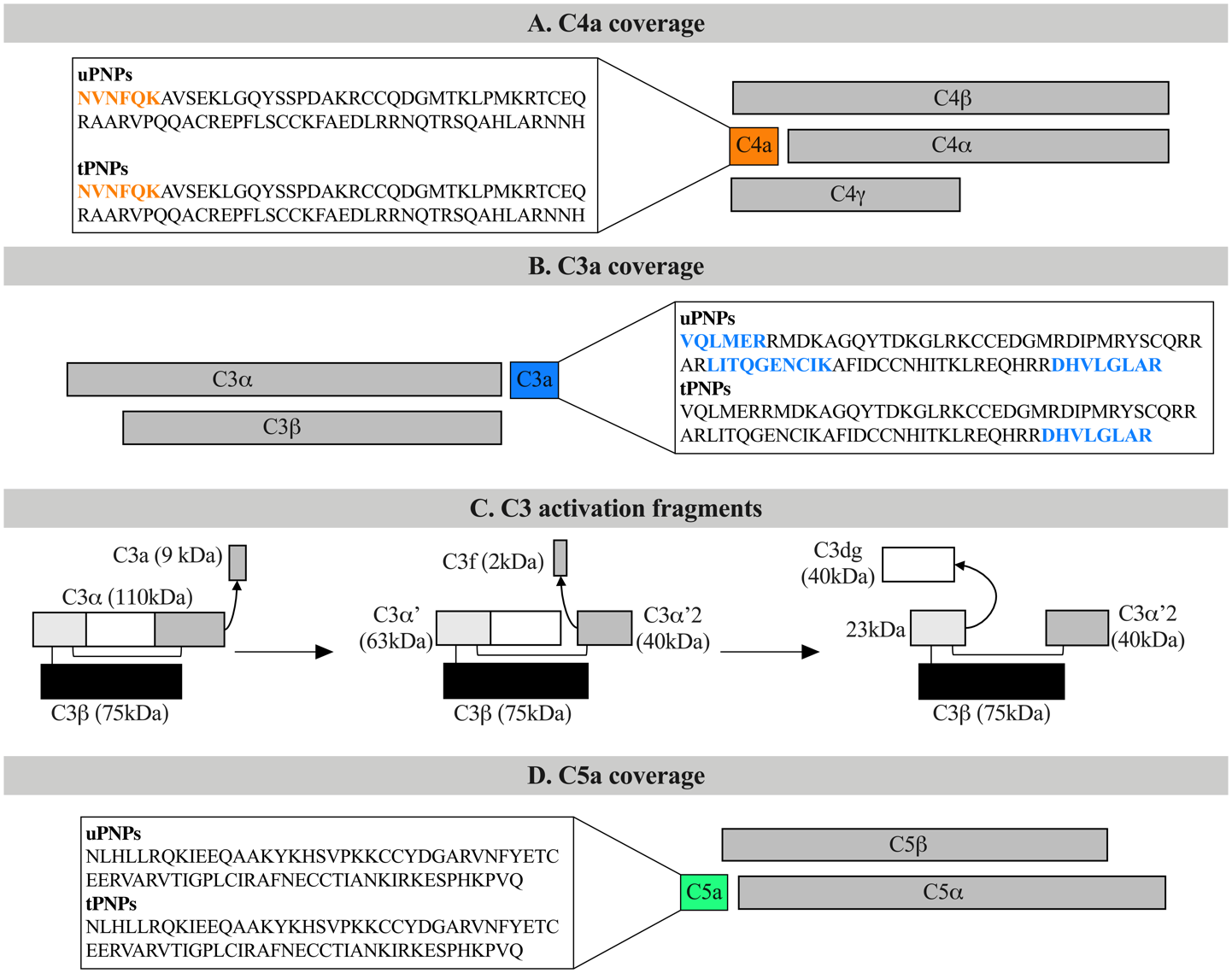
**

**Supplementary Figure 2. Complement activation through LC-MS/MS analysis.** Peptides obtained from the LC-MS/MS analysis were mapped on the sequence of C4, C3, and C5 to assess the presence of activating molecules. Peptides corresponding to C4a (**A**, orange amino acids) and C3a (**B**, blue amino acids) fragments were evidenced. (**C**) Graphical representation of C3 cleavage after complement activation. Peptide corresponding to C5a were not detected (**D**). PNPs: polymeric nanoparticles; uPNPs: untargeted PNPs; tPNPs: targeted PNPs.

**Additional File 5: Supplementary Figure 3**

**

**

**Supplementary Figure 3. Complement activation through LC-MS/MS analysis.** Peptides obtained from the LC-MS/MS analysis were mapped on the sequence of C4, C3, and C5 to assess the presence of activating molecules. Peptides corresponding to C4a (**A**, orange amino acids), C3a (**B**, blue amino acids) and C5a (**C**, blue amino acids). PNPs: polymeric nanoparticles; uPNPs: untargeted PNPs; tPNPs: targeted PNPs.
